# FGF21 upregulation by hepatitis C virus via the eIF2α-ATF4 pathway: implications for interferon signaling suppression and TRIM31-mediated TSC degradation

**DOI:** 10.3389/fmicb.2024.1456108

**Published:** 2024-08-15

**Authors:** Liang Liu, Masahiko Ito, Satoshi Sakai, Jie Liu, Kazuyoshi Ohta, Kenji Saito, Kenji Nakashima, Shinya Satoh, Alu Konno, Tetsuro Suzuki

**Affiliations:** ^1^Department of Microbiology and Immunology, Hamamatsu University School of Medicine, Shizuoka, Japan; ^2^Department of Molecular Biology, Hamamatsu University School of Medicine, Hamamatsu, Shizuoka, Japan; ^3^2nd Department of Internal Medicine, Hamamatsu University School of Medicine, Hamamatsu, Shizuoka, Japan

**Keywords:** hepatitis C virus, FGF21, ATF4, CREBH, TRIM31, SOCS2, ER stress

## Abstract

Hepatitis C virus (HCV) infection is a major cause of chronic liver diseases and is known to induce endoplasmic reticulum (ER) stress, which alters cellular homeostasis and metabolic processes. While ER stress is implicated in HCV-related diseases, its precise role remains unclear. This study identifies fibroblast growth factor 21 (FGF21) as a key host factor significantly upregulated by HCV infection. Mechanistic analyses reveal that the activation of the FGF21 promoter by HCV is primarily mediated by the transcription factor ATF4, which is upregulated through the phosphorylation of eIF2α induced by ER stress. Additionally, CREBH activation further enhances ATF4 expression, contributing to increased FGF21 levels. TRIB3, upregulated by ATF4, acts as a negative regulator of FGF21 expression. The study also identifies FGF21-dependent upregulation of SOCS2 and TRIM31 in HCV-infected cells. SOCS2 contributes to the suppression of type 1 interferon signaling, aiding viral persistence, while TRIM31 promotes the degradation of the tumor suppressor protein TSC, activating the mTORC1 pathway and potentially promoting liver cell proliferation. These findings suggest that FGF21 upregulation in HCV-infected cells may play a role in both immune response regulation and cell proliferation, contributing to sustained viral infection and disease progression.

## 1 Introduction

Hepatitis C virus (HCV) infection is highly persistent and carries the risk of lifelong disease, including cirrhosis and hepatocellular carcinoma (HCC). In addition to fibrosis and inflammation, frequent abnormalities in lipid and glucose metabolism are characteristic of the pathogenesis of chronic hepatitis C. Although safe, well-tolerated, and curable treatments for HCV infection have emerged in recent years, tens of millions of people still live with chronic HCV infection worldwide, with ~1.5 million new infections each year.

HCV is an enveloped virus with a positive-sense single-stranded RNA genome belonging to the Flaviviridae family. The precursor polyprotein of HCV, consisting of approximately 3,010 amino acids, is cleaved co- and post-translationally into 10 structural and non-structural (NS) components by cellular signal peptidases and viral proteases NS2 and NS3-4A, which are associated with the endoplasmic reticulum (ER) membrane. During the translation and processing of HCV proteins in infected hepatocytes, substantial rearrangement of the ER membrane architecture occurs, including the formation of double-membrane vesicles (DMVs), where viral genome replication takes place (Choi et al., 2018).

The assembly of infectious viral particles involves nucleocapsid formation, ER budding, and virion maturation, which has been shown to selectively recruit ER membranes around lipid droplets where viral structural proteins and viral replication complexes co-localize (Lee et al., 2019). It has also been reported that a topological map of how HCV coordinates the steps of viral replication and virion assembly was obtained by decorating the ER membranes surrounding lipid droplets with DMVs.

The ER is a dynamically interconnected membranous intracellular organelle where processes such as folding and cleavage of synthesized proteins, formation of disulfide bonds, and addition of glycans occur at the ER membrane surface and in the lumen. Changes in glycosylation, the presence of mutant proteins, oxidative stress, DNA damage, and other environmental factors can affect protein folding, leading to the accumulation of misfolded proteins. When protein folding function in the ER is impaired, homeostasis is disrupted, resulting in ER stress and the accumulation of unfolded proteins.

HCV infection has been shown to induce ER stress triggered by the localization of viral proteins to the lumen and membrane of the ER and ER-specific modifications in host cells, and the ER stress response may be involved in alterations in cellular homeostasis, including metabolic abnormalities. It has been reported that HCV infection or HCV protein expression induces ER stress in cells, leading to changes in the expression of cellular factors (Schmidt-Mende et al., 2001; García-Mediavilla et al., 2005; Pal et al., 2010; Sun et al., 2011; Yao et al., 2014; Chusri et al., 2016; Guo et al., 2019; Ríos-Ocampo et al., 2020).

For example, the c-Jun N-terminal kinase-dependent induction of ER stress by HCV infection has been shown to promote transforming growth factor beta1 (TGF-β1) expression (Chusri et al., 2016), and ER stress-induced activation of transcriptional regulators such as peroxisome proliferator-activated receptor gamma coactivator 1α in infected cells (Yao et al., 2014). Although the specific HCV proteins involved in ER stress induction differ according to previous reports, Core, NS2, NS3/4A, and NS5A have all been shown to be capable of inducing ER stress (García-Mediavilla et al., 2005; Pal et al., 2010; Sun et al., 2011; Ríos-Ocampo et al., 2020).

We have reported that ER stress induction by HCV infection or the expression of viral Core-NS2 contributes to the activation of the ER membrane-anchored cyclic adenosine monophosphate (AMP)-responsive element-binding protein H (CREBH), a liver-enriched transcription factor. Activated CREBH recognizes the transforming growth factor-beta2 (TGF-β2) promoter, enhancing TGF-β2 transcription (Chida et al., 2017), and upregulates the promoter of hepcidin, which acts on the iron transporter ferroportin to regulate cellular and plasma iron levels (Ohta et al., 2023). We have also provided evidence that HCV triggers the activation of the ER-associated degradation (ERAD) pathway, a key quality control mechanism responsible for the clearance of misfolded proteins in the ER for cytosolic proteasomal degradation, thereby contributing to the quality control of viral glycoproteins and virus particle production (Saeed et al., 2011).

Since ER stress is an intracellular environmental change that is generally induced by HCV infection, it may play a role in the development of viral pathogenesis. However, its relevance has not yet been fully elucidated.

In this study, we comprehensively identified host genes upregulated by HCV infection-induced ER stress, focusing on fibroblast growth factor 21 (FGF21), which is known to be involved in metabolic regulation (Tillman and Rolph, 2020). We elucidated the molecular mechanism of FGF21 gene expression regulation by HCV infection.

Further comprehensive comparative analysis of gene expression using FGF21 knockdown cells revealed for the first time that FGF21 may be involved in suppressing the interferon-dependent signaling pathway and promoting the tripartite motif containing 31 (TRIM31)-dependent degradation of the tumor suppressor protein tuberous sclerosis complex 2 (TSC2).

## 2 Materials and methods

### 2.1 Reagents

Mouse monoclonal antibody against HCV Core (2H9) was generated as described previously (Masaki et al., 2008). The following antibodies were used: mouse monoclonal antibodies against glyceraldehyde-3-phosphate dehydrogenase (GAPDH) (6C5, Santa Cruz Biotechnology, Dallas, TX) and CD81 (#555675, BD Biosciences, Franklin Lakes, NJ); rabbit monoclonal antibodies against phosphorylated eukaryotic initiation factor 2 alpha (elF2α) (#3398, Cell Signaling Technology, Danvers, MA), Phospho-p70 S6 kinase (#9234, Cell Signaling Technology), activating transcription factor 4 (ATF-4) (#11815, Cell Signaling Technology), tribbles pseudokinase 3 (TRIB3) (ab75846, Abcam, Cambridge, UK) and TSC2 (#4308, Cell Signaling Technology); and rabbit polyclonal antibodies against elF2α (#9722, Cell Signaling Technology), TRIM31 (PA5-116520, Thermo Fisher Scientific, Waltham, MA) and HCV NS3 (GTX131276, GeneTex, Irvine, CA) were used. Tunicamycin and interferon-α (IFNα) were obtained from Merck (Darmstadt, Germany) and Sumitomo Dainippon Pharma (Osaka, Japan), respectively.

### 2.2 Cell cultures

Human hepatoma HuH-7 derivative cell line Huh7.5.1, which is HCV negative and highly permissive for HCV infection (Masaki et al., 2010) (gifts from Dr. Francis V. Chisari, The Scripps Research Institute, La Jolla, CA, USA) and human embryonic kidney-derived HEK293T cells were maintained in Dulbecco's Modified Eagle Medium (DMEM) supplemented with non-essential amino acids, 100 U/ml of penicillin, 100 μg/ml of streptomycin, and 10% of fetal bovine serum at 37°C in a 5% CO_2_ incubator. CREBH-knockout (CREBH-KO) cells derived from Huh7.5.1 cells were produced using the CRISPR-Cas9 system as described (Ohta et al., 2023).

### 2.3 Plasmids

Expression plasmids for the HCV proteins pCAG-Core, -Core-NS2, and NS3-NS5B were previously described (Shi et al., 2016). Luciferase reporter plasmids carrying the human FGF21 promoter and its 5′-deletions (Uebanso et al., 2011) were generously provided by Professor Y. Taketani (University of Tokushima, Tokushima, Japan). These FGF21 promoter sequences were subcloned into pGL4.10 (Promega, Madison, WI, USA), resulting in plasmids F21-Luc1, F21-Luc2, and F21-Luc3, which were used in this study.

To construct FGF21 promoter reporters with substitution mutations at the recognition site for ATF4 (nt−412-TGCATCA; the transcription start point is numbered as nt 1) and peroxisome proliferator-activated receptor α (PPARα; nt−339-CCCACGG) (F21-Luc4 and F21-Luc5, respectively), mutated DNA fragments (TAAAGTA for F21-Luc4 and CAAATTG for F21-Luc5, with mutated sites underlined) were amplified by KOD Plus mutagenesis (Toyobo, Osaka, Japan), followed by insertion into pGL4.10. Primer sequences used are shown in Supplementary Table 1.

A series of luciferase reporter plasmids carrying the human TRIB3 promoter based on pGL3-basic (Promega) were generously provided by Professor H. Hayashi (Nagoya City University, Nagoya, Japan) (Ohoka et al., 2005). To create the reporter plasmid pGL-5xISG15 ISRE, which contains five copies of the IFN-stimulated response element (ISRE) upstream of the luciferase gene, the DNA fragment (5′-gagctcCAGTTTCGGTvTTCCCCAGTTTCGGTTTCCCCAGTTTC GGTTTCCCCAGTTTCGGTTTCCCCAGTTTCGGTTTCC Cctcgagggatcc-3′) and its complementary fragment were synthesized (Eurofins Operon, Tokyo, Japan) and inserted into pGL4.27 (Promega).

### 2.4 Virus infection and transfection

J6/JFH-1-derived HCV was prepared as described by Wakita et al. (2005). Naïve Huh7.5.1 cells were infected with HCV J6/JFH-1 at various multiplicities of infection (MOIs). DNA transfection was performed using Lipofectamine LTX (Thermo Fisher Scientific, Waltham, MA).

### 2.5 Gene silencing

Cells seeded in 12-well plates were transfected with siRNA (20 pmol) using the Lipofectamine RNAiMAX Regent (Thermo Fisher Scientific). siRNAs targeting FGF21, ATF4, or CREBH, as well as a non-targeting control siRNA, were purchased from Thermo Fisher Scientific.

To generate a stable cell line with knockdown of the FGF21 gene, lentiviral particles expressing short hairpin (sh)RNAs were produced using the ViraPower Lentiviral Packaging Mix (Thermo Fisher Scientific). Synthesized shRNA expression cassettes targeting FGF21B (5′-TGCTATGACATCTCCTCTTTATT) and a non-specific control (5′-TGGCGCGATAGCGCTAATAATTT) were respectively cloned into pLL3.7 (a gift from Dr. Luk Parijs; Addgene plasmid # 11795; [http://n2t.net/addgene:11795; RRID:Addgene_11795]). The resulting pLL3.7-shFGF21 or pLL3.7-shNC, along with pLP1, pLP2, and pLP/VSVG plasmids (ViraPower Lentiviral Packaging Mix; Thermo Fisher Scientific), were co-transfected into HEK293T cells. The culture supernatants containing lentiviruses were collected 48 h post-transfection and used for lentiviral delivery. After co-culturing with the virus mixture for 3 days, Huh7.5.1 cells expressing green fluorescence were selected by cell sorting, and FGF21 knockdown was confirmed in the collected cell clones. Cloned cells with high knockdown efficiency were used in this study.

### 2.6 RNA extraction and RT-quantitative PCR (qPCR)

RT-qPCR for mRNA quantification of cellular genes was performed as previously described (Liu et al., 2024). In brief, total cellular RNAs were isolated using the TRI reagent (Molecular Research Center, OH) and transcribed using the SuperScript VILO cDNA Synthesis Kit (Thermo Fisher Scientific). Aliquots of cDNAs were subjected to 40 cycles of PCR amplification. Quantitative qPCR was performed in the CFX Connect Real-Time System (Bio-Rad, Hercules, CA) using the THUNDERBIRD SYBR qPCR mix (Toyobo, Osaka, Japan). Briefly, reversed transcribed cDNAs together with forward and reverse primers were used for PCR. The thermal cycling conditions were as follows: 1 min at 95°C, followed by 40 cycles at 95°C for 15 sec and 60°C for 30 sec. RNA expression data were normalized to GAPDH levels using the comparative threshold cycle method. Primer sequences are shown in Supplementary Table 1.

To determine HCV RNA copies in cells, total RNAs isolated from cells were checked for concentration using a Nanodrop (Thermo Fisher Scientific), then diluted to 0.01 μg/μL, and aliquots were subjected to RT-PCR amplification. For particle-associated HCV RNA, culture supernatants were collected from infected cells and treated with PNE solution (8.45% PEG, 0.445 M NaCl, and 13 mM EDTA) for 1 h on ice. To remove free nucleic acids, pellets were incubated with RNase A for 1 h at 37°C (Invitrogen). After treatment with proteinase K (Nacalai Tesque) at 56°C overnight, RNA was isolated by phenol/chloroform extraction and ethanol precipitation. qRT-PCR was performed in the CFX Connect Real-Time System using TaqMan Fast Virus 1-Step Master Mix (Applied Biosystems).

### 2.7 Luciferase reporter assay

Cells seeded in a 24-well plate at a density of 5 × 10^4^ cells/well were transiently co-transfected with renilla luciferase and firefly luciferase vectors, with or without HCV infection or the viral protein expression. After 2 days, luciferase activities were measured using the Dual-Luciferase reporter assay system (Promega). Firefly luciferase activity was normalized to co-transfected renilla luciferase activity expressed from pGL4.75 (Promega).

### 2.8 Chromatin immunoprecipitation (ChIP) assay

According to the manufacturer's protocol, the ChIP assay was performed using the SimpleChIP^®^ Enzymatic Chromatin IP Kit (Cell Signaling Technology). Nuclear extracts were immunoprecipitated with a monoclonal anti-ATF4 antibody and normal rabbit IgG as a negative control. After the recovery of the DNA, qPCR was performed using two primer sets (one set is in nt −458 ~ −369, another set in nt −1918 ~ −1832) encompassing the human FGF21 promoter as shown in Supplementary Table 1.

### 2.9 Immunoblotting

Immunoblotting was performed essentially as previously described (Li et al., 2023). In brief, cell lysates were separated by SDS–PAGE and transferred onto polyvinylidene difluoride membranes. After blocking, membranes were incubated with a primary antibody overnight at 4°C. After washing, membranes were incubated with an HRP-conjugated secondary antibody (Cell Signaling Technology) for 1 h. Chemiluminescence was detected using the FUSION (Vilber Bio Imaging).

### 2.10 Enzyme-linked immunosorbent assay (ELISA)

Protein levels of FGF21 in cultured cells and their supernatants were determined using the Human FGF-21 Quantikine ELISA Kit (R&D Systems, Minneapolis, MN). Cell samples suspended in phosphate-buffered saline containing a protease inhibitor cocktail (Roche Diagnostics, Switzerland) were lysed by sonication. After centrifugation, the supernatants were subjected to ELISA.

### 2.11 Microarray analysis

mRNAs were labeled with Cy3 mono-reactive dye (for cell samples with HCV infection or tunicamycin treatment) and Cy5 mono-reactive dye (for control cell samples) dyes (GE Healthcare, Little Chalfont, UK) and purified according to the manufacturer's protocol (3DGENE mRNA CyDye label v2, Toray Industries, Tokyo, Japan). The concentration of labeled RNAs was determined using a NanoDrop 1000 spectrophotometer (Thermo Fisher Scientific) before hybridization onto microarray chips (Human Oligo Chip 25k ver. 2.10., Toray Industries). Hybridization and subsequent washing were performed according to the manufacturer's protocol (3DGENE mRNA hybridization v2, Toray Industries). The intensity of labeled mRNAs was analyzed with the 3D-Gene Scanner 3000 system (Thermo Fisher Scientific).

### 2.12 RNA sequencing (RNA-seq)

Huh7.5.1-based FGF21-knockdown cells and control cells introducing shFGF21 or shNC, respectively, were infected with or without HCV J6/JFH-1 (MOI = 0.5) and collected samples at 72 h post-infection (hpi). The quality and quantity of total cellular RNAs isolated by the TRI reagent were assessed using the UV absorbance ratio. RNA-seq was performed by Macrogen Japan using the manufacturer's reagents and protocol. Trimmed reads were mapped to the reference genome using HISAT2, a splice-aware aligner (Kim et al., 2015). Individual transcripts were assembled by StringTie (Pertea et al., 2015) with aligned reads, providing information on known transcripts, novel transcripts, and alternative splicing transcripts. A human reference genome library GRCh38 was used to map cDNA fragments obtained from RNA-seq data. Statistical analysis was performed using Fold Change (Klymenko et al., 2017) and edgeR (Robinson et al., 2010) for each paired transcript to be compared.

### 2.13 Statistical analysis

Values are presented as means from triplicate experiments along with their standard deviations (SD). Statistical analyses were primarily conducted using Analysis of Variance (ANOVA), followed by Tukey's HSD test for *post-hoc* pairwise comparisons between groups. A significance threshold of p < 0.05 was used to identify statistically significant differences.

## 3 Results

### 3.1 Comprehensive analysis of host gene upregulation by HCV-induced ER stress

To comprehensively identify host genes significantly upregulated by HCV infection-induced ER stress, we conducted the following analyses. Huh7.5.1 cells were infected with the HCV J6/JFH-1 strain and harvested at 48- and 72-h post-infection. As a reference for stress induction, cells treated with tunicamycin, a known inducer of ER stress through inhibition of glycoconjugate synthesis, were also prepared. Gene expression in HCV-infected, tunicamycin-treated, and untreated cells was analyzed using a cDNA microarray. Among the 16,320 human genes detected, those with expression increases of 2.5-fold or more at 48 h and 5-fold or more at 72 h post-HCV infection, as well as those with similar induction by tunicamycin, were selected and summarized (Table 1).

**Table 1:** Host factors induced by HCV infection identified by microarray analysis.

**Gene_ symbol**	**Gene function**	**TM treatment**			**HCV infection (48 hpi)**			**HCV infection (72 hpi)**			**Expression in normal liver (nTPM)**
		**Cy5_TM-**	**Cy3_TM**+	**Ratio (Cy3/Cy5)**	**Cy5_ 48N**	**Cy3_ 48HCV**	**Ratio (Cy3/Cy5)**	**Cy5_ 72N**	**Cy3_ 72HCV**	**Ratio (Cy3/Cy5)**	
INHBE	Inhibin subunit beta E chain To inhibit the secretion of follitropin by the pituitary gland	49.0	433.3	8.8	18.7	186.8	10.0	39.5	1,186.3	30.1	144.2
TNFRSF9	TNF receptor superfamily To enhance immune response and T-cell activation	18.2	50.3	2.8	4.6	42.4	9.3	4.0	143.0	35.4	0.7
ATF3	Activating transcription factor To regulate stress response, apoptosis, and cellular proliferation	41.0	127.9	3.1	39.5	161.7	4.1	51.8	777.1	15.0	135.7
TMEM140	Predicted to be an integral component of the membrane	78.8	355.8	4.5	55.8	160.2	2.9	73.9	571.6	7.7	52.0
BBC3	BCL2 binding component To promote apoptosis, involved in the p53-mediated cell death pathway	168.5	418.1	2.5	83.8	240.3	2.9	164.4	1,225.9	7.5	5.4
FGF21	Fibroblast growth factor To regulate glucose metabolism, insulin sensitivity, and energy homeostasis	6.7	19.2	2.9	3.7	9.2	2.5	4.4	30.9	7.1	82.1

Huh7.5.1 cells with or without HCV infection (MOI = 0.5) were collected at 48- and 72-h post-infection (hpi). The cells with tunicamycin (TM) treatment were prepared as a reference. mRNAs were labeled with Cy3 mono-reactive (for cell samples with HCV infection and TM treatment) and Cy5 mono-reactive (for control cell samples) dyes. cDNA microarray analysis yielded a total of 24,267 data points. After eliminating incomplete data, we narrowed it down to 16,320 data points for further analysis. The expression of each gene, indicated as normalized fluorescence intensity values, was listed. The ratio of C3-labeled intensity to C5-labeled intensity was calculated. Selection criteria for filtering included: (1) a ratio (value at 48 hpi to the non-infected group) > 2.5; (2) a ratio (value at 72 hpi to the non-infected group) > 5; (3) a ratio (with TM to without TM) > 2.5. Finally, six genes were indicated in the table. The expression level of each gene in normal liver tissue (normalized transcripts per million; nTPM) obtained from the Human Protein Atlas (https://www.proteinatlas.org/) was also shown.

**Figure 1:**
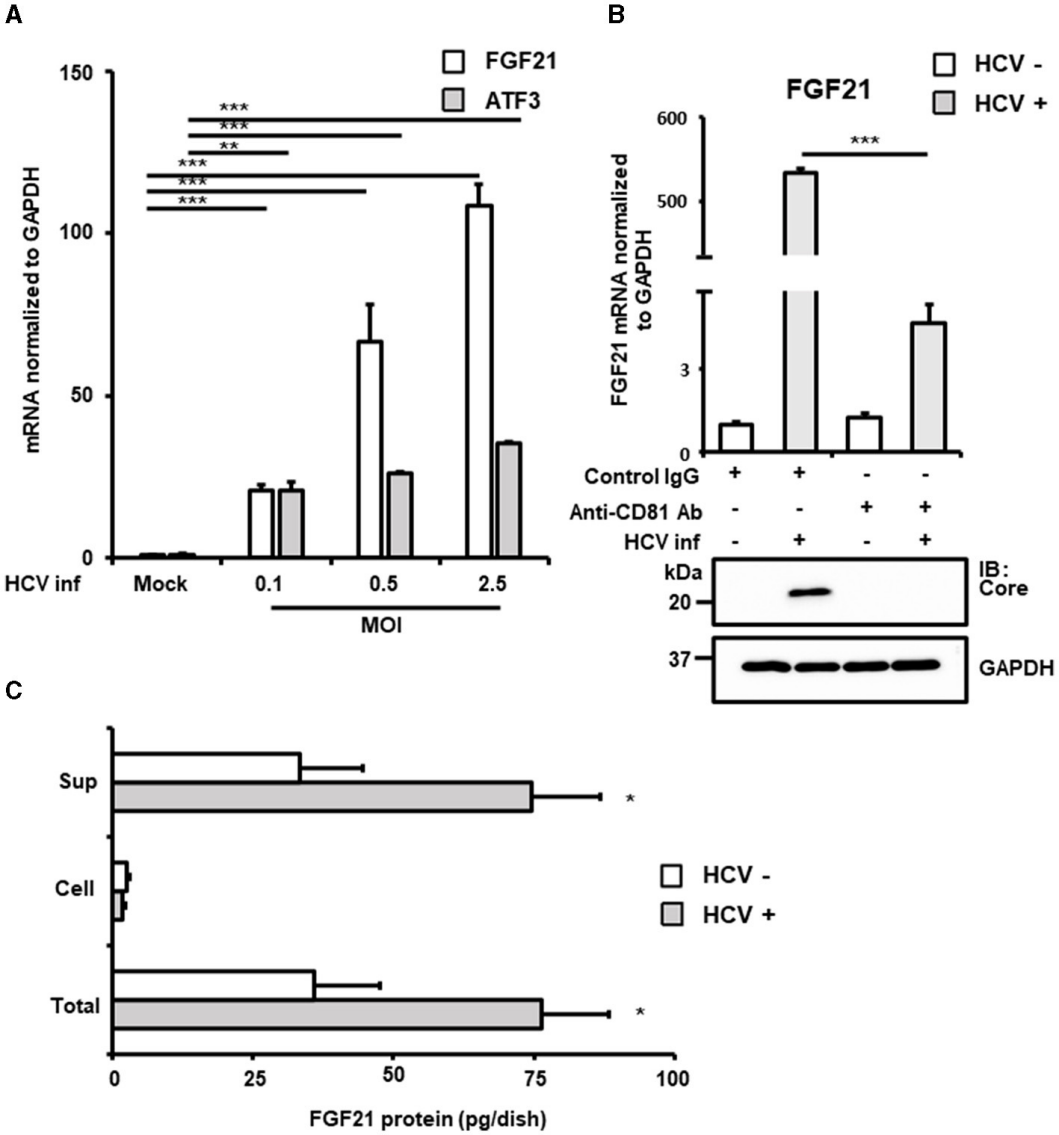
Increased expression of FGF21 in cells infected with HCV. **(A)** Huh7.5.1 cells infected with HCV J6/JFH-1 (MOI = 0, 0.1, 0.5, and 2.5). Cell lysates were prepared at 2.5 days post-infection (dpi). mRNA expression levels of FGF21, ATF3, and GAPDH were determined by RT-qPCR. **(B)** The cells were pretreated with anti-CD81 antibody or control mouse-IgG for 1 h before HCV infection (MOI = 0.5). FGF21 mRNA was quantified at 3 dpi. HCV Core and GAPDH were analyzed by immunoblotting. **(C)** Cell lysates and culture supernatants from HCV-infected cells (MOI = 0.5) were prepared at 3 dpi, subjecting to the determination of FGF21 protein levels per 10 cm dish by ELISA. Results are presented as means ± SD (*n* = 3). A statistical analysis was conducted using ANOVA with *post hoc* Tukey's test for pairwise group comparisons. *p < 0.05; **p < 0.01; ***p < 0.001.

From the identified genes, activating transcription factor 3 (ATF3) and fibroblast growth factor 21 (FGF21) were selected due to their high expression in normal liver tissues and potential implications in HCV-related metabolic abnormalities and carcinogenesis. RT-qPCR analysis confirmed that both mRNAs were upregulated in a virus titer-dependent manner, with FGF21 showing a particularly pronounced increase, exceeding 100-fold over uninfected cells at 72 h post-infection (Figure 1A, Supplementary Figure 1). Thus, this study attempted to investigate the mechanisms of FGF21 upregulation in HCV-infected cells.

FGF21, a key regulator of energy homeostasis primarily produced in the liver, showed significantly increased mRNA expression upon HCV infection. This increase was significantly inhibited by pretreatment with anti-CD81 antibody, confirming that HCV infection induced FGF21 expression (Figure 1B). ELISA quantification of FGF21 protein levels in cell lysates and culture supernatants of both HCV-infected and uninfected cells revealed that most FGF21 was secreted into the culture supernatants. FGF21 protein levels in both supernatants and total culture (including intracellular portions) increased with the extent of HCV infection (Figure 1C).

The induction of FGF21 mRNA expression by HCV infection was significantly suppressed by pretreatment of cells with the anti-CD81 antibody (Figure 1B). However, a moderate induction of FGF21 expression was also observed in the CD81 antibody-treated group. This suggests that even when cells are pretreated with an excessive amount of antibodies, it is difficult to block the infection pathway completely, allowing a small number of viruses to enter the cells and proliferate. It is assumed that a certain amount of ER stress is induced during this process, leading to the induction of a low level of FGF21 expression.

### 3.2 Identification of regulatory factors for FGF21 activation by HCV infection

**Figure 2:**
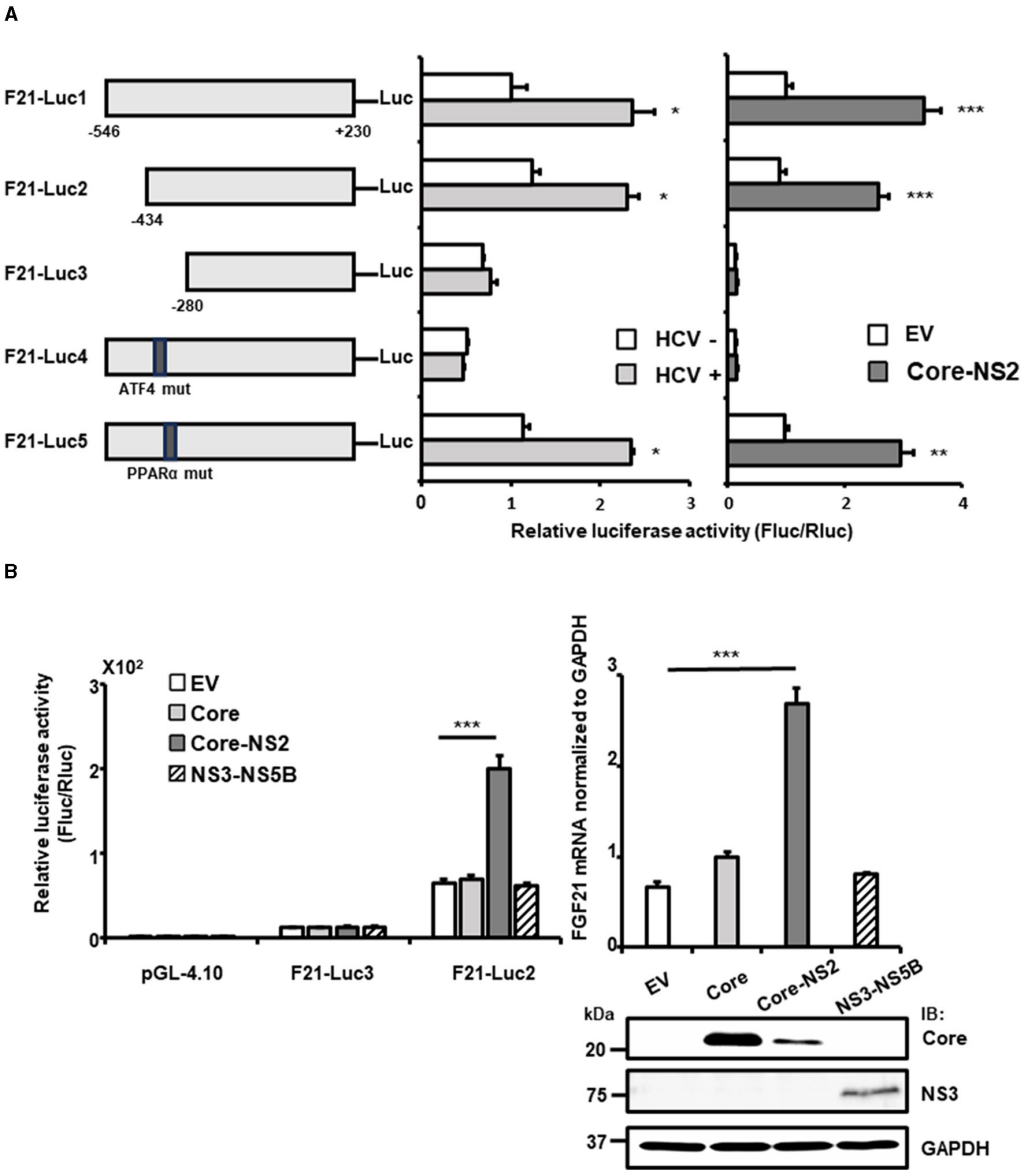
Determination of the region/sequence in the FGF21 promoter is important for its upregulation induced by HCV infection and the viral protein expression. **(A)** A schematic representation of the FGF21 promoter reporter carrying the firefly luciferase (Luc) gene and its mutated constructs (F21-Luc1, -Luc2, -Luc3, -Luc4, and -Luc5) is shown. Substitution mutations to the consensus sequence of ATF4 (nt−413-TTAAAGTA) and PPARα (nt−341-GGCAAATTGCCA; mutated nt are underlined) were introduced into F21-Luc4 and F21-Luc5, respectively. HuH-7.5.1 cells transfected with either reporter plasmid, together with HCV infection (MOI = 0.5) or with cotransfection with an HCV protein-expressing plasmid (Core-NS2) or an empty vector (pCAGGS; EV), were prepared at 2 days post-transfection (dpt), followed by luciferase assay. Luc activities normalized by Renilla luciferase activities were determined as relative luciferase activity. **(B)** Left: FGF21 promoter reporters (F21-Luc2 and F21-Luc3) or a basal reporter (pGL4.10) was transfected into cells together with an HCV protein-expressing plasmid (Core, Core-NS2, or NS3-NS5B) or an EV. Cells were prepared at 2 dpt, and Luc activities were normalized by Renilla, and luciferase activities were determined. Right: Cells transfected with an HCV protein-expressing plasmid (Core, Core-NS2, or NS3-NS5B) or an empty vector (EV) were prepared at 3 dpt. Cellular mRNA levels of FGF21 were determined by RT-qPCR. Core, NS3, and GAPDH were analyzed by immunoblotting. Results are presented as means ± SD (*n* = 3). A statistical analysis was conducted using ANOVA with *post hoc* Tukey's test for pairwise group comparisons. *p < 0.05; **p < 0.01; ***p < 0.001. Core, NS3, and GAPDH were analyzed by immunoblotting.

To identify the regulatory regions and sequences involved in the transcriptional activation of FGF21 by HCV infection, a promoter-reporter assay was performed (Figure 2A, left and middle). Partial deletion analysis of the FGF21 promoter region (F21-Luc1, F21-Luc2, and F21-Luc3) indicated that a 155 bp region from nt −280 to −434 upstream of the transcription start site is crucial for the activation of the FGF21 promoter by HCV infection. This region contains recognition sites for ATF4 and PPARα, both of which are known to regulate FGF21 transcription.

When point mutations were introduced into these recognition sequences, the mutation in the PPARα site (F21-Luc5) had little effect on promoter activity. In contrast, the mutation in the ATF4 site (F21-Luc4) reduced promoter activity to its basal level, eliminating the enhancement observed with HCV infection. Further analysis showed that FGF21 promoter activity and FGF21 mRNA levels were most significantly increased by the expression of the HCV Core-NS2 polyprotein, with minimal effects from Core alone or NS3-NS5B expression (Figure 2B).

With the introduction of the Core-NS2 expression plasmid, the precursor polyprotein is expressed, leading to cleavage by ER-associated peptidases into Core, E1, E2, p7, and NS2 proteins. In the case of NS3-NS5B expression, it is expressed as a precursor polyprotein and then processed by NS3-4A protease into NS3, NS4A, NS4B, NS5A, and NS5B. Deletion of the nt−280 to−434 region (F21-Luc3) and mutation of the ATF4 recognition sequence (F21-Luc4) abolished the inductive effect of HCV protein expression, indicating that ATF4 plays a critical role in FGF21 upregulation induced by HCV, particularly via the viral proteins generated from the Core-NS2 polyprotein.

**Figure 3:**
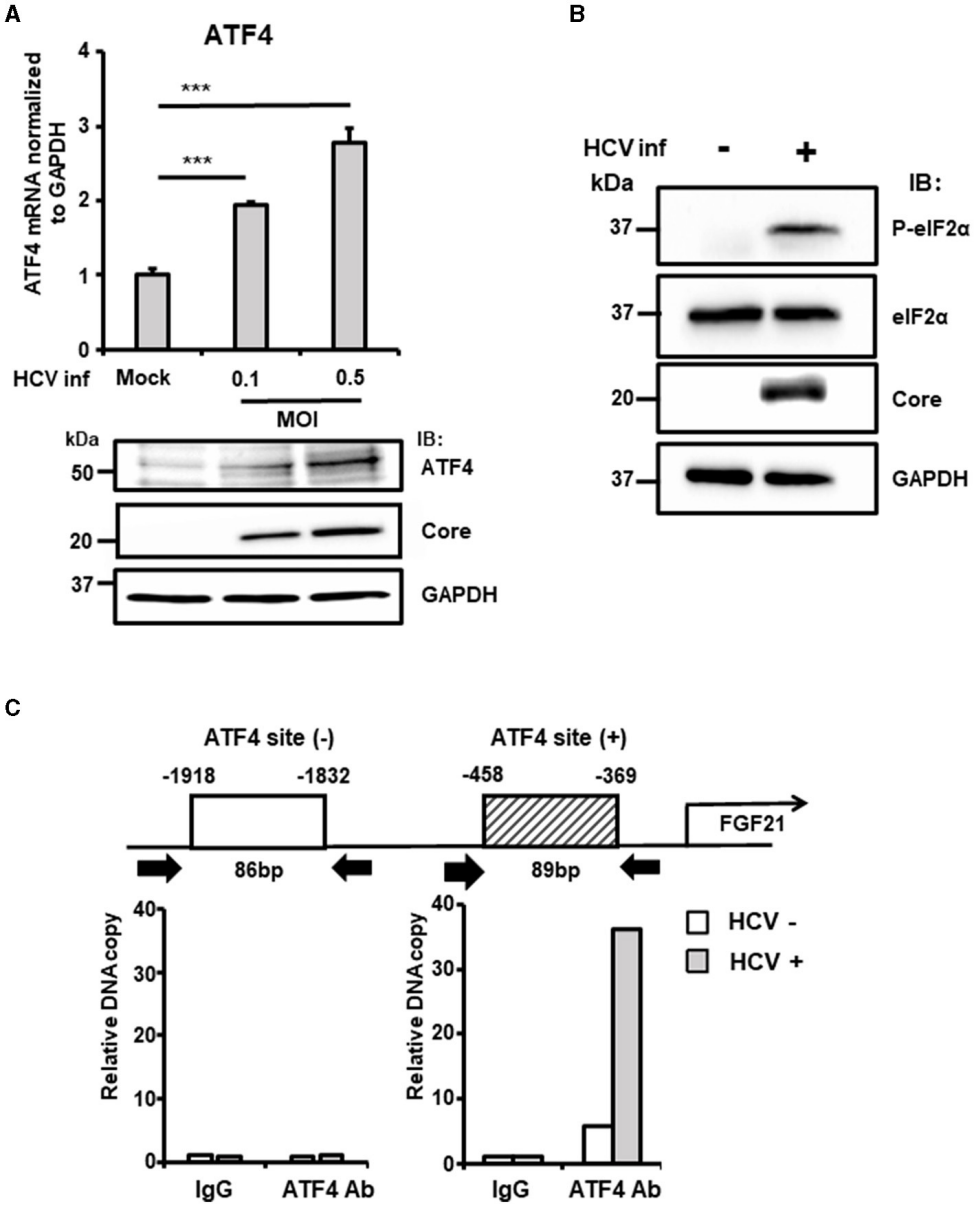
The critical role of ATF4 in the upregulation of FGF21 expression induced by HCV. **(A)** Huh7.5.1 cells infected with HCV J6/JFH-1 (MOI = 0, 0.1, and 0.5) were prepared at 3 dpi. mRNA levels of ATF4 in cells were determined by RT-qPCR. ATF4, Core, and GAPDH were analyzed by immunoblotting. **(B)** Cells infected without or with HCV (MOI = 0.5) were prepared at 3 dpi. Phosphorylated (P-)elF2α, total elF2α, Core, and GAPDH were analyzed by immunoblotting. **(C)** At 3 dpi with or without HCV, cells were prepared, and nuclear fractions were used for ChIP assay. Protein-DNA complexes were immunoprecipitated either with an antibody against either ATF4 or with control rabbit IgG. Precipitates were analyzed by RT-qPCR. Results are presented as means ± SD (*n* = 3). A statistical analysis was conducted using ANOVA with *post hoc* Tukey's test for pairwise group comparisons. ***p < 0.001.

ATF4 is a stress-inducible transcription factor and a key indicator of integrated stress responses, including ER stress. Analysis revealed that ATF4 mRNA and protein levels increased in an HCV infection titer-dependent manner (Figure 3A). ER stress enhances the phosphorylation of eIF2α, a subunit of the translation initiation factor group, which, in turn, promotes the translation of specific factors, including ATF4. Consistent with this, HCV infection did not alter the total level of eIF2α protein but significantly increased the steady-state level of phosphorylated eIF2α (Figure 3B).

ChIP assays were performed to investigate the recruitment of transcription factors to the FGF21 promoter in the nucleus. DNA fragments containing the ATF4-binding region identified in previous experiments were amplified, showing significantly higher DNA copy levels in HCV-infected cells compared to uninfected cells.

In contrast, amplification of a region upstream of the FGF21 promoter that does not contain the ATF4 recognition site showed no difference between HCV-infected and uninfected cells (Figure 3C).

**Figure 4:**
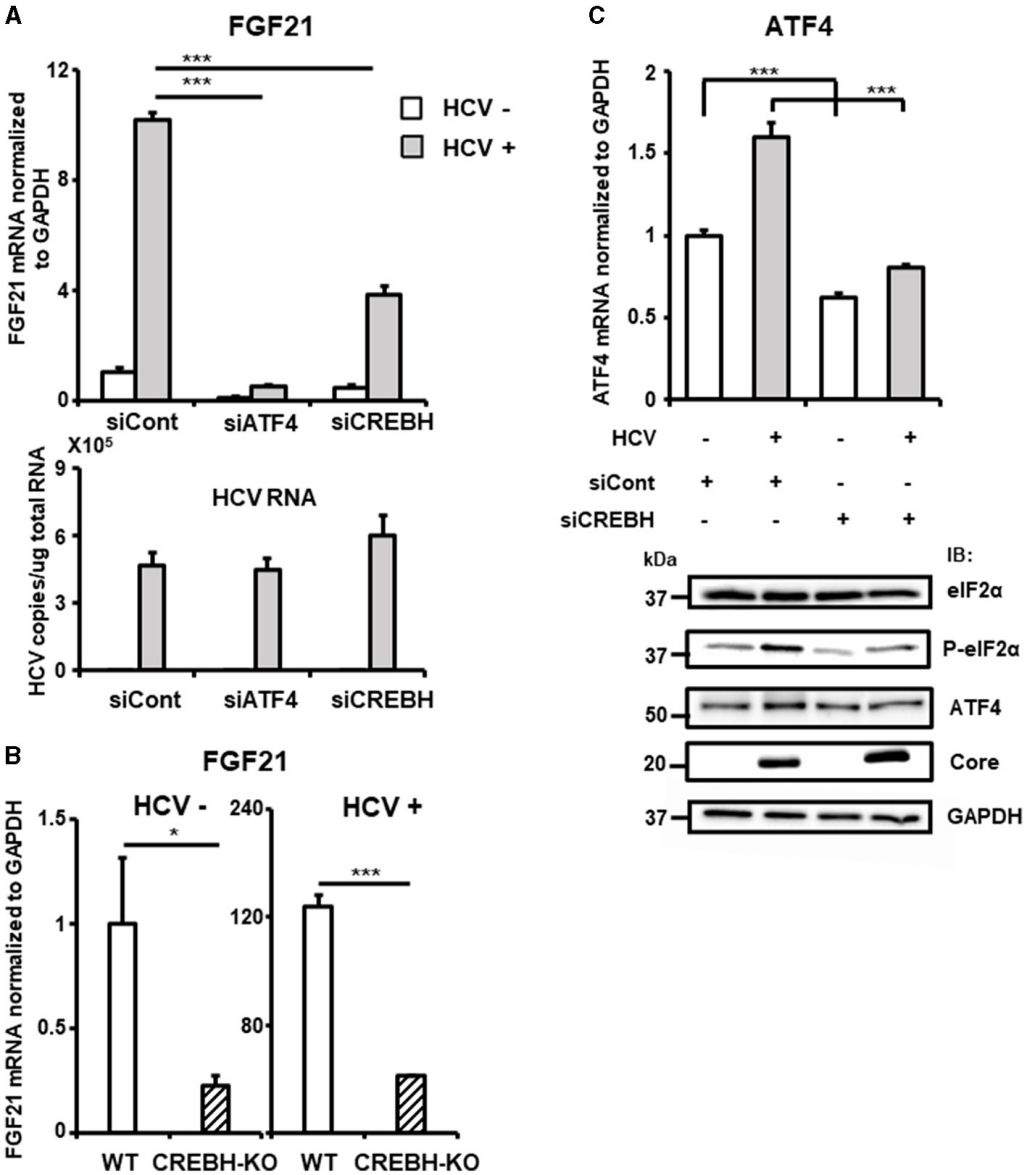
Involvement of CREBH in FGF21 upregulation induced by HCV infection. **(A)** Huh7.5.1 cells transfected with siRNA against ATF4 or CREBH or negative control (NC) siRNA together with HCV infection (MOI = 0.5) were prepared at 3 dpi. mRNA levels of FGF21, ATF4, CREBH, and HCV RNA copies in siRNA-treated cells were determined by RT-qPCR. **(B)** CREBH-KO and parental Huh7.5.1 cells with or without HCV infection were prepared at 3 dpi. mRNA levels of FGF21 in cells were determined by RT-qPCR. **(C)** Cells transfected with siRNA against CREBH or NC siRNA together with HCV infection were prepared at 3 dpi. mRNA levels of ATF4 were determined by RT-qPCR. P-elF2α, total elF2α, ATF4, Core, and GAPDH were analyzed by immunoblotting. Results are presented as means ± SD (*n* = 3). A statistical analysis was conducted using ANOVA with *post hoc* Tukey's test for pairwise group comparisons. *p < 0.05; ***p < 0.001.

Knockdown of the ATF4 gene by siRNA almost completely abolished the induction of FGF21 mRNA expression by HCV infection without altering the cells' susceptibility to HCV infection (Figure 4A, Supplementary Figure 2). These results suggest that ER stress caused by HCV infection leads to the phosphorylation of eIF2α and increased ATF4 expression, which, in turn, promotes ATF4 recruitment to the FGF21 promoter, leading to increased FGF21 expression.

The liver-specific transcription factor CREBH also contributes to the upregulation of FGF21 transcription and is implicated in the control of hepatic steatosis. We have demonstrated that HCV induces CREBH activation in infected cells (Chida et al., 2017). Knockdown of the CREBH gene by siRNA significantly reduced the induction of FGF21 expression by HCV, though the reduction was moderate compared to that of ATF4 knockdown (Figure 4A, Supplementary Figure 2). CREBH knockout (CREBH-KO) cells, generated using the CRISPR-Cas9 system, showed that FGF21 mRNA levels were approximately one-quarter of those in parental cells, both with and without HCV infection (Figure 4B). It remains unclear how the eIF2α-ATF4 signaling pathway, a key mechanism activated via the integrated stress response, is involved in CREBH activation. Knockdown of CREBH suppressed the HCV-induced increase in ATF4 expression and eIF2α phosphorylation (Figure 4C), suggesting that CREBH activation triggered by HCV infection plays a role in upregulating ATF4 expression through eIF2α phosphorylation, thereby contributing to the upregulation of FGF21 transcription.

**Figure 5:**
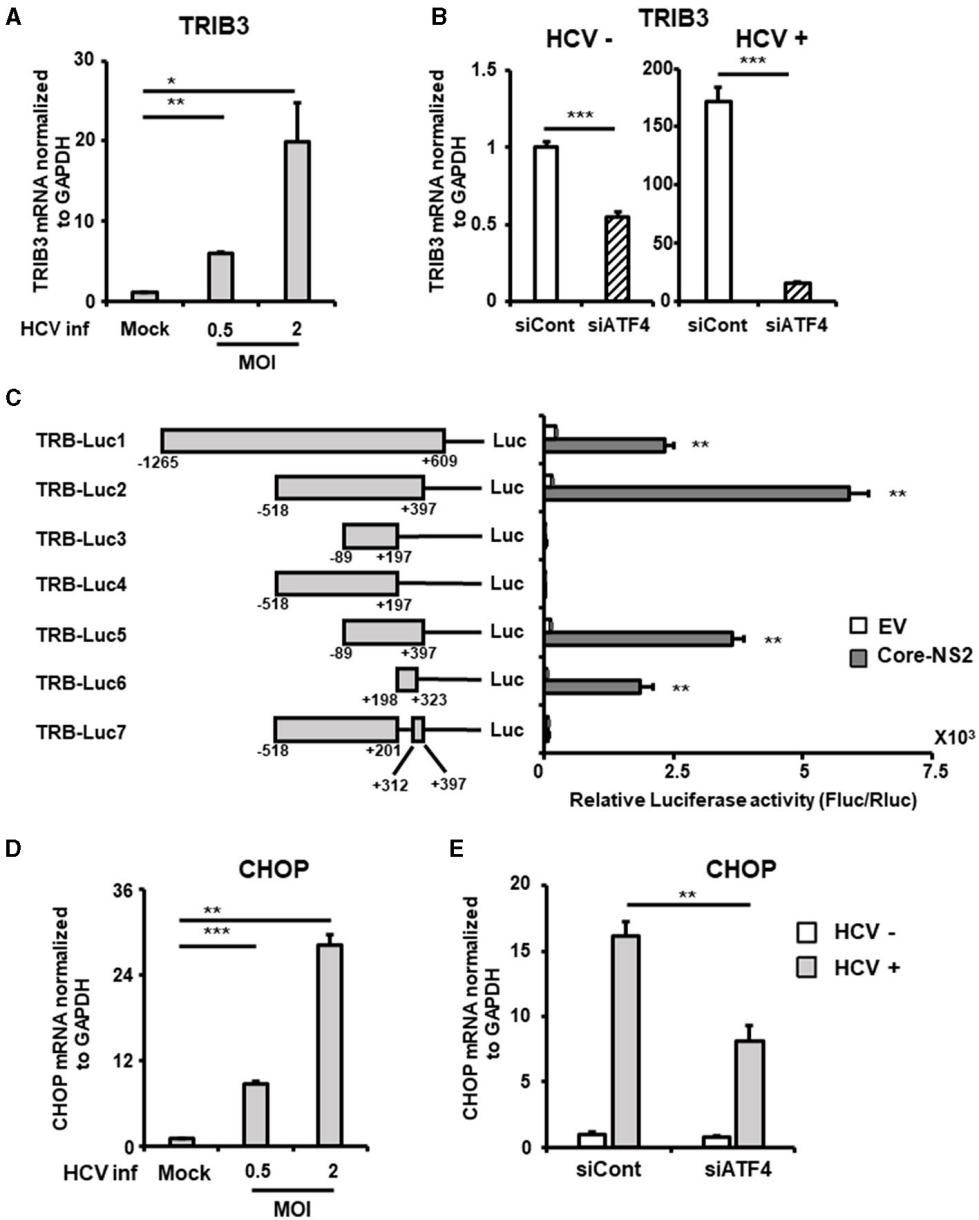
Involvement of ATF4/CHOP in TRIB3 upregulation induced by HCV infection. **(A)** Huh7.5.1 cells infected with HCV (MOI = 0, 0.5, and 2) were prepared at 2.5 dpi. mRNA levels of TRIB3 in cells were determined by RT-qPCR. **(B)** Cells transfected with siRNA against ATF4 or NC siRNA together with HCV infection (MOI = 0.5) were prepared at 3 dpi. TRIB3 mRNA levels were determined by RT-qPCR. **(C)** Schematic representation of the TRIB3 promoter reporters carrying the firefly luciferase (Luc) gene (TRB-Luc1, -Luc2, -Luc3, -Luc4, -Luc5, -Luc6, and -Luc7) is shown. Cells transfected with TRIB3 promoter reporter together with an HCV Core-NS2-expressing plasmid (Core-NS2) or an empty vector (EV) were prepared at 2 dpi. Luc activities normalized by Renilla luciferase activities were determined as relative luciferase activity. **(D)** Cells infected with HCV (MOI = 0, 0.5, and 2) were prepared at 2.5 dpi. CHOP mRNA level was determined by RT-qPCR. **(E)** Cells transfected with siRNA against ATF4 or NC siRNA together with HCV infection (MOI = 0.5) were prepared at 3 dpi, followed by the determination of CHOP mRNA levels by RT-qPCR. Results are presented as means ± SD (*n* = 3). A statistical analysis was conducted using ANOVA with *post hoc* Tukey's test for pairwise group comparisons. *p < 0.05; **p < 0.01; ***p < 0.001.

TRIB3 (also known as TRB3), a molecule whose expression is controlled by ATF4, is highly expressed in the liver and acts as a regulator of the integrated stress response. Although TRIB3 has been reported to negatively regulate FGF21 promoter activity under nutrient deprivation conditions, its relevance to viral infection is unknown. TRIB3 mRNA levels increased in an HCV infection titer-dependent manner (Figure 5A). This HCV-induced increase in TRIB3 expression was significantly reduced by ATF4 knockdown (Figure 5B, Supplementary Figure 3A).

**Figure 6:**
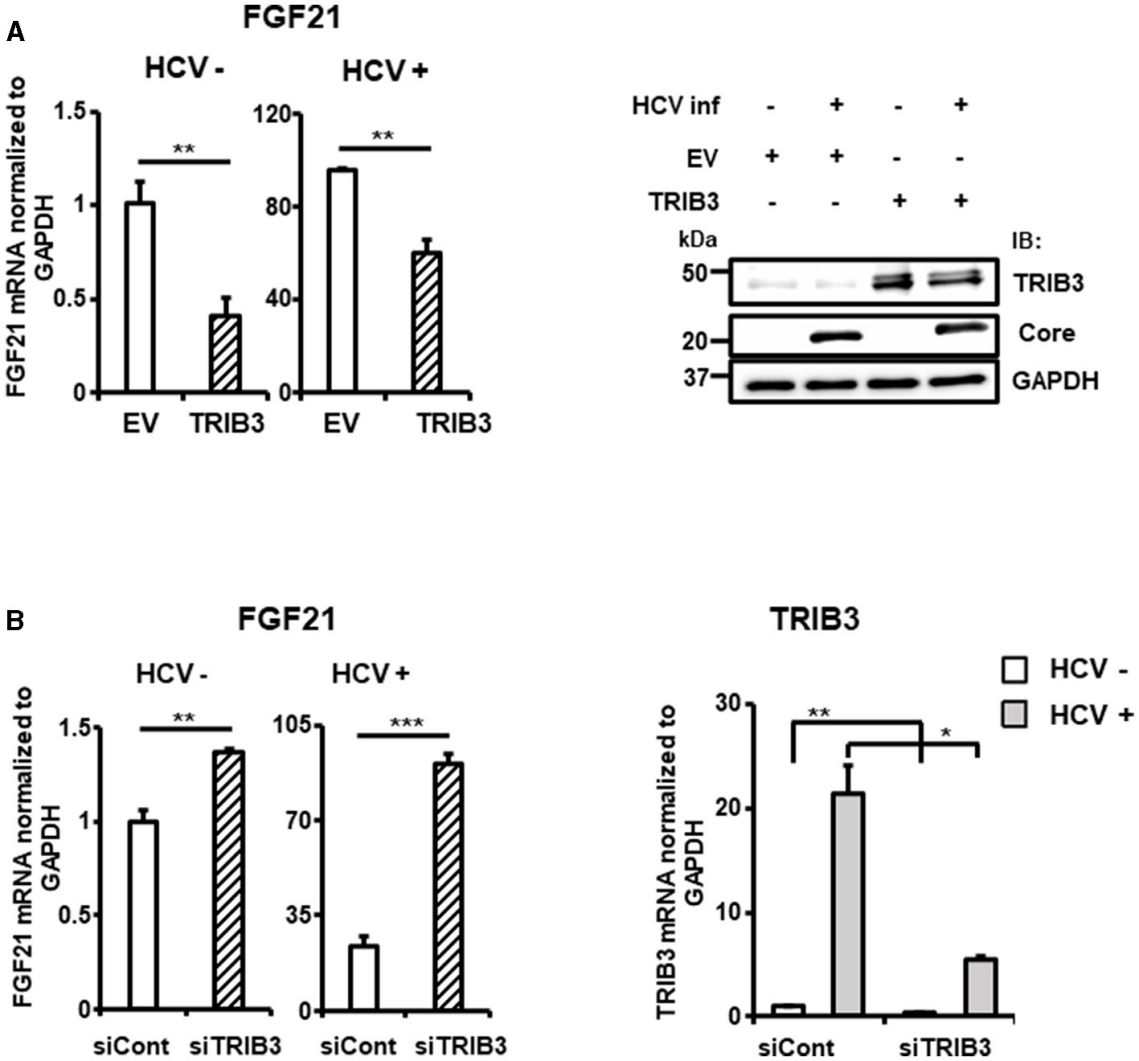
Suppression of FGF21 expression by TRIB3, which is upregulated by HCV. **(A)** Huh7.5.1 cells transfected with TRIB3-expression plasmid or an empty vector (EV) together with HCV infection were prepared at 3 dpi. FGF21 mRNA levels were determined by RT-qPCR. Protein expression of TRIB3, HCV Core, and GAPDH in cells prepared was analyzed by immunoblotting. **(B)** Cells transfected with siRNA against TRIB3 or NC siRNA together with HCV infection were prepared at 3 dpi. mRNA levels of FGF21 and TRIB3 were determined by RT-qPCR. Results are presented as means ± SD (*n* = 3). A statistical analysis was conducted using ANOVA with *post hoc* Tukey's test for pairwise group comparisons. *p < 0.05; **p < 0.01; ***p < 0.001.

To elucidate the molecular mechanism of TRIB3 transcriptional activation by HCV Core-NS2 expression, a promoter-reporter analysis was performed using a series of partial deletions (Figure 5C). TRIB3 promoter activity was largely retained in TRB-Luc6 but completely lost in TRB-Luc3 and TRB-Luc4, indicating that the region from nt +201 to +312 is crucial for increased activity due to expression of the viral proteins. This region contains three ATF4/CHOP recognition sequences (Supplementary Figure 3B), and both ATF4 and CHOP were shown to be upregulated by HCV infection (Figures 3A, 5D), suggesting that the increased expression of both transcription factors contributes to TRIB3 transcriptional activation. Furthermore, ATF4 knockdown significantly reduced CHOP expression in HCV-infected cells, indicating that ATF4 regulates CHOP expression (Figure 5E, Supplementary Figure 3C). We then investigated whether increased or decreased expression of TRIB3 affects FGF21 mRNA expression. Transfection of uninfected cells with the TRIB3 expression vector led to a reduction in FGF21 mRNA levels, and high expression of TRIB3 also decreased FGF21 mRNA expression in HCV-infected cells (Figure 6A). Conversely, knockdown of TRIB3 resulted in increased levels of FGF21 mRNA regardless of HCV infection status. Notably, FGF21 mRNA levels were enhanced up to threefold by TRIB3 knockdown in HCV-infected cells (Figure 6B), suggesting that TRIB3 potentially contributes to the negative regulation of FGF21 expression induced by HCV infection.

### 3.3 Identification of cellular genes that are expressed in an FGF21-dependent manner upon HCV infection

FGF21 is known for its role in various metabolic regulations, but its full physiological functions are not completely understood. Considering the possibility that FGF21, which is induced by HCV infection, may contribute to persistent HCV infection and pathogenic expression, we aimed to identify host factors involved in these processes that are FGF21-dependent. We established a stable FGF21-knockdown cell line by transducing Huh7.5.1 cells with lentiviral vectors carrying shRNA targeting FGF21. The corresponding control cell line was generated with non-targeted shRNA (shNC). Using these cell lines, we identified genes significantly upregulated by HCV infection and downregulated by FGF21 knockdown through RNA-seq analysis. Genes fitting these criteria, involved in immune regulation or cell proliferation, and expressed at significant levels in the normal liver are summarized in Table 2. Among these, suppressor of cytokine signaling 2 (SOCS2) and TRIM31 were analyzed separately due to their likely involvement in persistent viral infection and liver disease development.

**Table 2:** Genes of interest selected from RNA-seq analyses.

**Gene symbol**	**shNC/cont (TPM)**	**shNC/HCV (TPM)**	**shFGF21/cont (TPM)**	**shFGF21/HCV (TPM)**	**shNC/HCV vs. shNC/cont**	**shFGF21/cont vs. shNC/cont**	**shFGF21/HCV vs. shNC/HCV**	**Expression in normal liver (nTPM)**	**Function**
					**Fold induction (FDR** ***p*****-value)**	**Fold change (FDR** ***p*****-value)**	**Fold change (FDR** ***p*****-value)**		
HDAC9	0.9	11.7	0.3	0.5	12.9 (0.000)	0.3 (0.251)	0.04 (0.000)	2.8	To play a role in transcriptional regulation, cell cycle progression, and developmental events
SOCS2	0.4	3.0	0.1	0.9	7.1 (0.006)	0.3 (0.025)	0.3 (0.158)	57.3	To regulate growth hormone signaling, impacting growth and immune response modulation
CREB5	4.0	24.3	0.7	9.3	6.0 (0.004)	0.2 (0.000)	0.4 (0.207)	1.8	To be involved in regulating gene expression and cellular growth processes
APLF	0.8	3.8	0.9	0.8	4.9 (0.020)	1.1 (0.702)	0.2 (0.044)	1.5	To contribute to DNA repair and cell cycle regulation
IFIH1	0.9	4.3	0.3	0.8	4.8 (0.022)	0.4 (0.176)	0.2 (0.029)	8.3	Sensor of viral RNAs that trigger the innate immune response
RARB	3.4	14.2	1.3	0.8	4.2 (0.029)	0.4 (0.182)	0.1 (0.000)	5.8	To regulate cell growth and differentiation via retinoic acid signaling
TRIM31	7.6	31.7	6.2	4.8	4.1 (0.019)	0.8 (0.993)	0.2 (0.013)	1.1	Ubiquitin ligase is involved in the regulation of the mTORC pathway and immune response
GEM	1.8	7.3	0.3	2.5	4.1 (0.043)	0.2 (0.032)	0.3 (0.227)	19.7	Playing a crucial role in cell shape and motility.

RNA sequencing was performed on four groups of samples, including the control group with or without HCV infection and the FGF21 knockdown group with or without HCV infection. The expression level of each gene was denoted by transcripts per million (TPM). The three sets of ratios used for screening are shown as follows. (Exp 1) shNC/HCV vs. shNC/cont: Huh7.5.1 cells introducing negative control shRNA (shNC) with vs. without HCV infection (MOI = 0.5 for 72 h infection). (Exp 2) shFGF21/cont vs shNC/cont: the cells with shFGF21 vs. shNC. (Exp 3) shFGF21/HCV vs. shNC/HCV: HCV-infected cells with shFGF21 vs. shNC. Eight genes whose expression was upregulated 4-fold or more by HCV infection in Exp 1 and significantly downregulated by FGF21 knockdown in Exp 2 or Exp 3 (p-value: < 0.05) were listed in the table. Three sets of ratios for the gene expression were also shown. The expression level of each gene in normal liver tissue (normalized transcripts per million; nTPM) obtained from the Human Protein Atlas was indicated.

**Figure 7:**
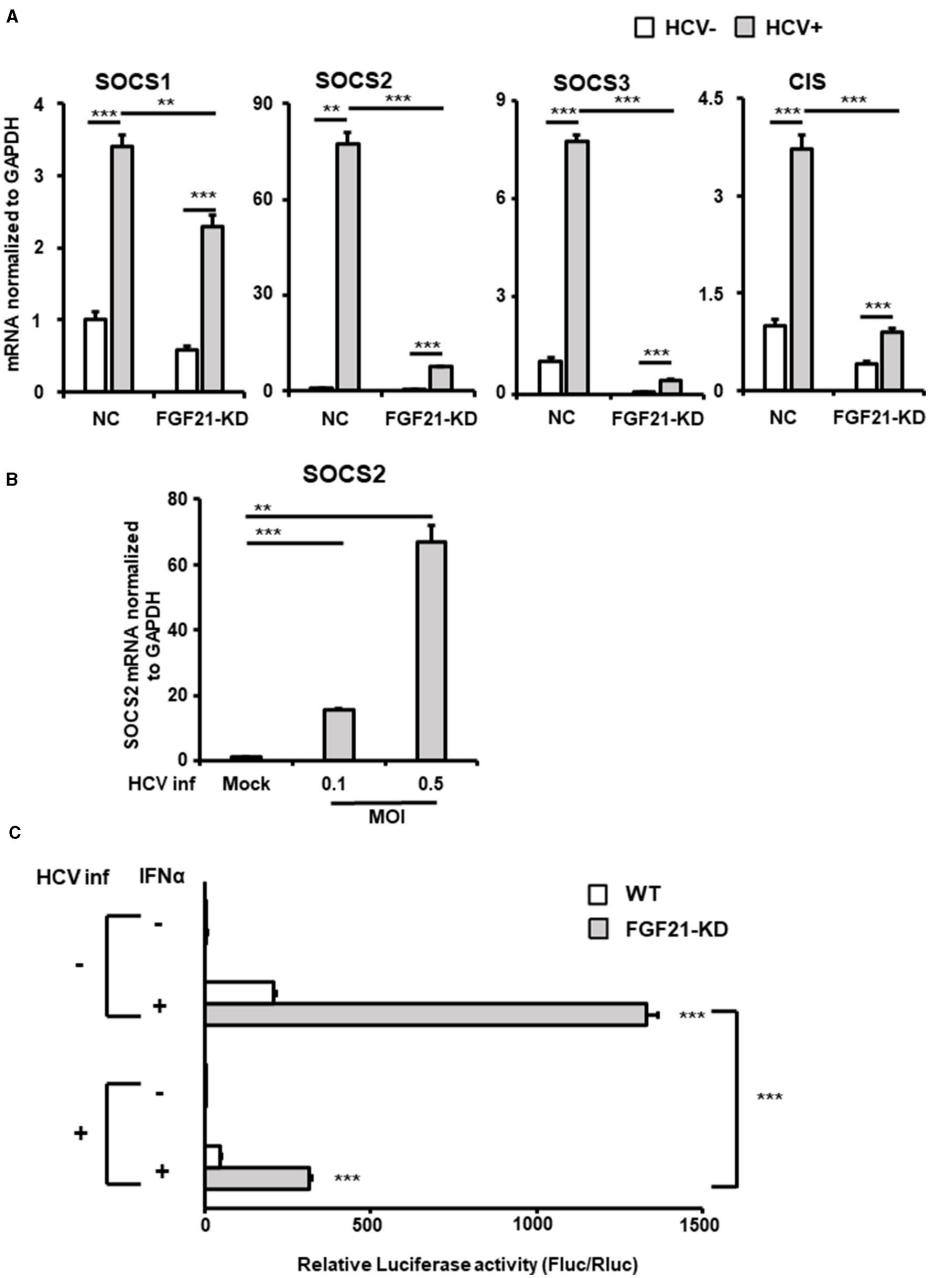
Involvement of FGF21 in the negative regulation of IFN signaling through induction of SOCS2 expression caused by HCV infection. **(A)** Huh7.5.1 cells introducing shFGF21 (FGF21-KD) or shNC (NC) were cultured with or without HCV infection for 3 days, followed by determination of mRNA levels of SOCS1, SOCS2, SOCS3, and CIS by RT-qPCR. **(B)** Cells infected with HCV (MOI = 0, 0.1, and 0.5) were prepared at 2.5 dpi. SOCS2 mRNA levels were determined by RT-qPCR. **(C)** FGF21-KD and parental Huh7.5.1 cells with or without HCV infection were transfected with pGL4.27-based ISRE-containing reporter and pGL4.75 (Renilla luciferase reporter) 1 dpi. One day later, IFNα (300 IU/ml) was added to the cells, followed by harvesting the cells at 2.5 dpi. Luc activities normalized by Renilla luciferase activities were determined as relative luciferase activity. Results are presented as means ± SD (*n* = 3). A statistical analysis was conducted using ANOVA with *post hoc* Tukey's test for pairwise group comparisons. **p < 0.01; ***p < 0.001.

SOCS2, part of the SOCS protein family, acts as a feedback inhibitor of the JAK-STAT pathway by interacting with JAK (Janus kinase) or STAT (Signal Transducer and Activator of Transcription) proteins to inhibit signaling and regulate cellular cytokine responses. Among the eight members of the SOCS family, SOCS1, SOCS2, SOCS3, and cytokine-inducible SH2-containing protein (CIS) are known to be particularly involved in the repression of the JAK-STAT pathway and are expressed in the liver. RT-qPCR analysis showed that SOCS2 was the most significantly upregulated among the SOCS family members upon HCV infection, increasing ~75-fold compared to < 8-fold for SOCS1, SOCS3, and CIS (Figure 7A). This induction of SOCS2 expression was dramatically reduced by FGF21 knockdown (Figure 7A, Supplementary Figure 4). SOCS2 mRNA levels increased in an HCV titer-dependent manner (Figure 7B).

The binding of IFNs to type 1 IFN receptor activates the JAK-STAT pathway, which, in turn, activates the transcription of many IFN-stimulated genes by binding the transcription factor complex Interferon-Stimulated Gene Factor 3 to the ISRE sequence in the nucleus. Luciferase reporter plasmids containing ISRE were introduced into FGF21-knockdown and control cells, and reporter activity was compared with and without HCV infection and IFN stimulation (Figure 7C). In the uninfected state, ISRE reporter activity increased approximately 200-fold with IFN in control cells and about 490-fold in FGF21 knockdown cells. The induction of ISRE activity by IFN was attenuated by HCV infection, as previously reported (Kanazawa et al., 2004), likely due to SOCS proteins suppressing ISRE activity. This suggests that FGF21 negatively regulates IFN signaling through SOCS2 upregulation.

**Figure 8:**
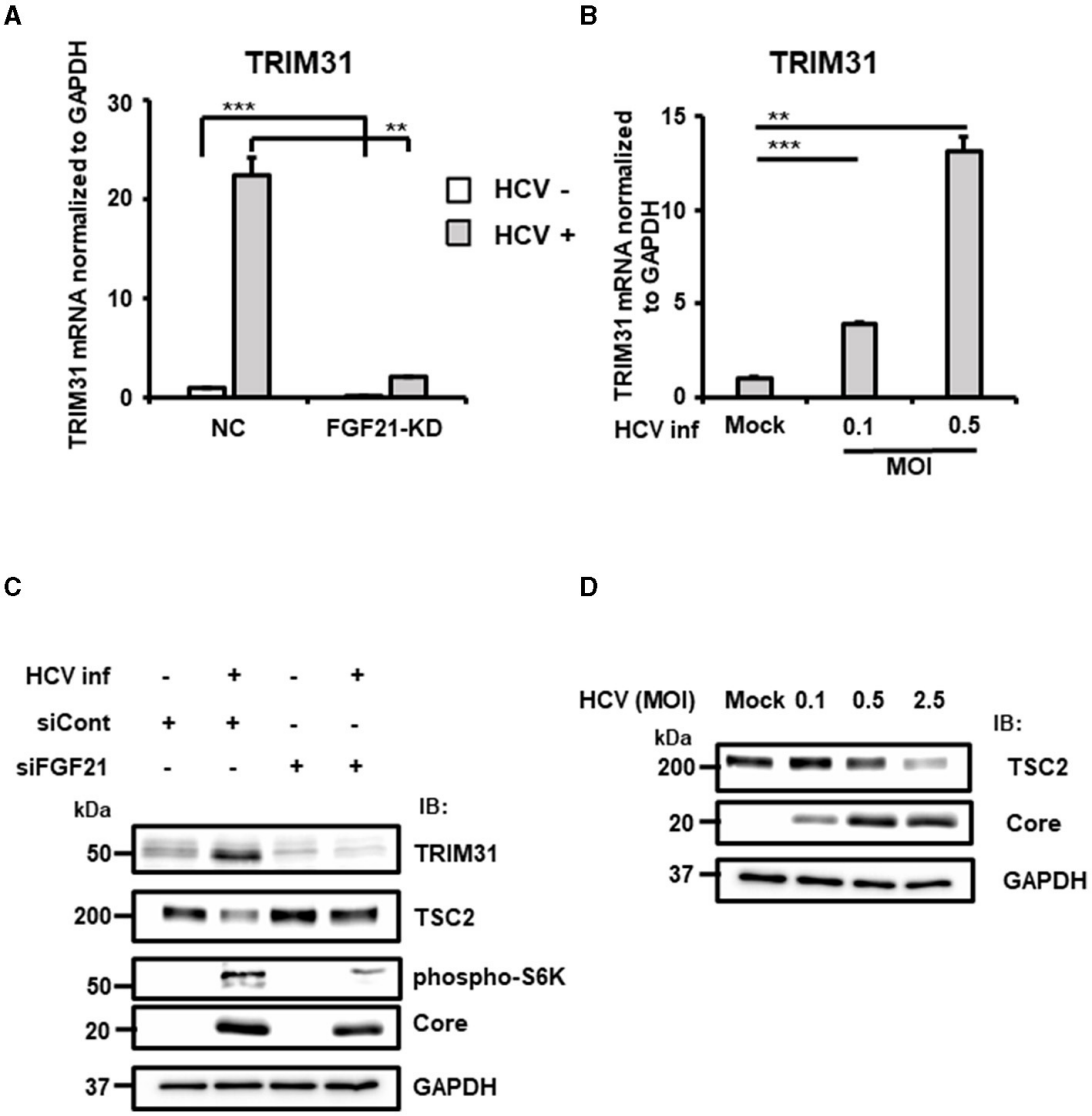
Involvement of FGF21 in the negative regulation of TSC protein level through induction of TRIM31 expression caused by HCV infection. **(A)** FGF21-KD- and negative control (NC) Huh7.5.1 cells were cultured with or without HCV infection for 3 days, followed by determination of TRIM31 mRNA levels by RT-qPCR. **(B)** Cells infected with HCV (MOI = 0, 0.1, and 0.5) were prepared at 3 dpi. TRIM31 mRNA levels were determined by RT-qPCR. Results are presented as means ± SD (*n* = 3). A statistical analysis was conducted using ANOVA with *post hoc* Tukey's test for pairwise group comparisons. **p < 0.01; ***p < 0.001. **(C)** Cells transfected with siRNA against FGF21 or siRNA control with or without HCV infection were prepared at 2.5 dpi. Protein expression of TRIM31, TSC2, phosphorylated p70 S6K, Core, and GAPDH were analyzed by immunoblotting. **(D)** Cells infected with HCV (MOI = 0, 0.1, 0.5, and 2.5) were prepared at 2.5 dpi. Protein expression of TSC2, HCV Core, and GAPDH was analyzed via immunoblotting.

TRIM31, an E3 ubiquitin ligase of the TRIM family, is associated with cancer, immune diseases, and metabolic dysfunction. It is implicated in degrading the TSC1 and TSC2 complex, which is a critical negative regulator of the mammalian target of rapamycin complex1 (mTORC1), promoting HCC progression by over-activating mTORC1 signaling (Guo et al., 2018). RT-qPCR confirmed that HCV-induced TRIM31 mRNA expression was largely canceled by FGF21 knockdown (Figure 8A). TRIM31 mRNA levels were upregulated by HCV infection in a titer-dependent manner (Figure 8B). Correspondingly, TRIM31 protein levels increased significantly with HCV infection, which was abolished by FGF21 knockdown. Contrarily, TSC2 protein expression was reduced by HCV infection in control cells but not in FGF21 knockdown cells (Figure 8C). Phosphorylated p70 S6K, a major effector in the mTORC1 network, was enhanced by HCV infection, with this increase reduced by FGF21 knockdown (Figure 8C).

Higher HCV infection levels were associated with lower TSC2 expression (Figure 8D). To explore whether the decrease in TSC2 protein levels was due to changes in TSC2 mRNA levels, RT-qPCR analysis was performed. Surprisingly, TSC2 mRNA levels in HCV-infected cells were ~2.5-fold higher than in uninfected cells (Supplementary Figure 5). This suggests that the decrease in TSC2 protein induced by HCV infection is most likely due to proteolysis, which is driven by the increased E3 ligase activity associated with increased TRIM31 expression. These results suggest that HCV infection and FGF21 negatively regulate TSC protein levels through the induction of TRIM31.

## 4 Discussion

The hepatokine FGF21, an atypical member of the fibroblast growth factor family, is a versatile metabolic regulator that acts in an endocrine manner and responds to various physiological and pathological stresses. Several transcription factors have been identified that regulate FGF21 expression under different pathophysiological conditions (Yang et al., 2023).

In particular, PPARα and ATF4 have been shown in several studies to activate the FGF21 promoter. It has been shown that a PPARα response element (PPRE) is present in the promoter region of the mouse and human FGF21 gene and that PPARα binds directly to the FGF21 promoter to promote its transcription (Inagaki et al., 2007; Lundåsen et al., 2007).

PPARα is a transcription factor that is closely involved in metabolic regulation and is normally activated during energy deprivation. Fasting activation of PPARα has been shown to affect circulating levels of FGF21 in the body (Gälman et al., 2008). PPARγ has also been shown to act as a transcription factor for FGF21 (Zhou et al., 2016).

ATF4, a member of the leucine zipper superfamily, is involved in various cellular responses to environmental stresses, intracellular perturbations, and growth factors. It has been shown that mitochondrial dysfunction (Kim et al., 2013) and treatment with transcriptional coactivator with PDZ-binding motif activator (Jung et al., 2015) enhance FGF21 mRNA expression by activating the eIF2α-ATF4 signaling pathway. Under ER stress, ATF4 binds to the FGF21 promoter and promotes its transcription. Three response elements to ATF4, amino acid response element (AARE)1, AARE2, and AARE3, have been shown to exist in the promoter region of the FGF21 gene (Maruyama et al., 2016).

The CREBH-FGF21 axis is known to be involved in the control of hepatic steatosis. It has been reported that the activation of CREBH by neutral lipid accumulation increases FGF21 mRNA expression and that FGF21 expression is decreased in CREBH-deficient mice (Nakagawa and Shimano, 2018; Hwang et al., 2023). The nuclear receptor superfamily, including retinoic acid receptor and retinoic acid receptor-related receptor α, has also been reported to regulate FGF21 transcription by binding to their respective response elements (Wang et al., 2010; Li et al., 2013).

Our promoter assay with partial deletions and substitution mutations in the FGF21 promoter indicated that ATF4 plays an important role in the activation of the FGF21 promoter by HCV infection and expression of Core-NS2 protein (Figure 2A). During the HCV life cycle, viral protein maturation involves the cleavage of precursor polyprotein and post-cleavage complex formation that are closely associated with the ER membrane and lumen. Thus, several HCV proteins associated with the ER, including Core, E1, E2, p7, and NS2, likely contribute to stress induction in infected cells.

The substitution mutation at the ATF4 recognition site used (F21-ATF4mut) contains mutations in AARE1 but not in AARE2 and AARE3. Although mutants of AARE2, AARE3, and nuclear receptor response elements were not analyzed, it is highly unlikely that transcription factors are recruited to sites other than AARE1 on the FGF21 promoter for FGF21 upregulation by HCV. This is because FGF21 induction was almost completely abolished by F21-ATF4mut.

The basal and HCV-induced levels of FGF21 expression were significantly reduced by knockdown or knockout of the CREBH gene (Figures 4A, B). HCV-induced phosphorylation of eIF2α and the enhancement of steady-state levels of ATF4 were abolished by CREBH knockdown (Figure 4C). However, the region important for HCV-induced expression, nt −434 ~ −280, as indicated in the promoter deletion assay, does not appear to contain a CREBH recognition site sequence. These findings suggest that CREBH activation by HCV infection leads to the upregulation of the eIF2α-ATF4 pathway, contributing to increased binding of ATF4 to the FGF21 promoter and enhanced FGF21 transcription.

In general, the integrated stress response begins with the activation of multiple eIF2α phosphatases corresponding to various stress pathways, culminating in eIF2α phosphorylation. Figure 4A shows that ATF4 knockdown almost completely abolishes HCV-induced FGF21 upregulation, whereas FGF21 induction remains to some extent with CREBH knockdown. This suggests that the activation of the eIF2α/ATF4 pathway from stress responses mediated by a factor(s) other than CREBH may also be involved in the induction of FGF21 expression from HCV infection.

TRIB3 is one of the target genes of ATF4 and is known as a negative feedback regulator of the ATF4-dependent transcription. It also participates in the regulation of the integrated stress response (Ohoka et al., 2005). It is of interest that the deletion of TRIB3 increases FGF21 levels in mouse liver during essential amino acid deficiency and in cultured cells during glucose starvation.

TRIB3 inhibits ATF4-binding dependent transcriptional activation of FGF21 at its promoter (Örd et al., 2018; Lu et al., 2024). In this study, we showed that HCV infection induces TRIB3 expression in an ATF4-dependent manner (Figure 5B) and that high TRIB3 expression suppresses FGF21 expression (Figure 6A).

The induction of ER stress by infection is known for many viruses, including HCV, and ER stress is generally considered to be a degenerative process involved in the pathogenesis of infectious diseases. To the best of our knowledge, this study is the first to demonstrate that a host factor induced by ER stress in virus-infected cells can act as a negative feedback regulator for another stress-inducible factor. The suppression of FGF21 induction by increased expression of TRIB3 in HCV infection may serve to prevent rapid changes in the cellular environment by inhibiting FGF21 intervention in various physiological pathways, such as metabolic control, cell proliferation, and immune response (see below) in infected cells, thus contributing to sustained viral infection.

The liver is a major organ that directs the endocrine FGF21 signaling pathway, and FGF21 acts to suppress the episodes of abnormal lipid and glucose metabolism and promote metabolic homeostasis in the liver. Primarily from animal studies, it has been shown that FGF21 does not directly regulate insulin or appetite but promotes lipid catabolism, including lipolysis, fatty acid oxidation, and mitochondrial oxidative activity (Sui and Chen, 2022; Yang et al., 2023).

To date, research on FGF21 has focused primarily on its beneficial functions in metabolic disorders such as obesity, type 2 diabetes, and non-alcoholic- or metabolic dysfunction-associated steatohepatitis by reducing fat mass, hyperglycemia, and dyslipidemia (Tillman and Rolph, 2020). Pharmacological administration of FGF21 to animals clearly has an ameliorative effect on metabolic abnormalities. However, it is also known that elevated levels of endogenous circulating FGF21 are associated with worsening metabolic status in human and animal experimental models (Keuper et al., 2020). It has been reported that FGF21 levels increase with obesity, insulin resistance, type 2 diabetes, and non-alcoholic fatty liver disease. In obese mice, exogenous FGF21 administration attenuates signaling responses and reduces metabolic improvement effects (Fisher et al., 2010; Chen et al., 2022).

Such a phenomenon is defined as FGF21 resistance, and it is considered that obesity is associated with resistance to FGF21. While some studies suggest that such FGF21 resistance may result from decreased expression of FGF receptor (FGFR) complexes (Hale et al., 2012), others have reported cases that do not depend on changes in the expression of such receptor-related factors (Markan et al., 2017). Thus, the molecular mechanisms of FGF21 resistance in obesity and metabolic disorders remain unclear.

In this study, comprehensive mRNA expression analyses (cDNA microarray and RNA-seq) and RT-qPCR revealed that FGF21 is one of the host-cell factors whose expression is markedly induced by HCV infection (Tables 1, 2, Figure 1). Additionally, the level of FGF21 protein secreted into the culture supernatant significantly increased in the presence of HCV infection (Figure 1C). The induction of FGF21 mRNA expression by HCV infection was significantly suppressed by THE pretreatment of cells with anti-CD81 antibody (Figure 1B). Nevertheless, a moderate induction of FGF21 expression was also observed in the CD81 antibody-treated group. This suggests that even when cells are pretreated with an excessive amount of antibodies, it is difficult to block the infection pathway completely, allowing a small number of viruses to enter the cells and proliferate.

It is assumed that a certain amount of ER stress is induced during this process, leading to the induction of a low level of FGF21 expression.

Since FGF21 is involved in the regulation of lipid and glucose metabolism, it may play a role in the pathogenesis of HCV-related diseases. We hypothesize that a condition similar to obesity-induced FGF21 resistance may be induced by persistent HCV infection, which, in turn, results in the functional decline of the FGF21 endocrine system to maintain metabolic homeostasis, leading to the metabolic abnormalities frequently observed in hepatitis C patients. We plan to investigate whether circulating FGF21 levels tend to be higher in patients with hepatitis C compared to other viral hepatitis cases.

The significance of FGF21 in oncogenesis has recently been highlighted; high levels of FGF21 have been observed in a variety of cancers (Sui and Chen, 2022), indicating its potential as a biomarker for cancer diagnosis and treatment. The close relationship between the liver and FGF21 has provided a deeper understanding of the FGF21-HCC axis compared to other cancers. Both serum and hepatic levels of FGF21 are elevated in patients with hepatitis, hepatic cirrhosis, and HCC (Huang et al., 2006). Additionally, elevated levels of FGF21 have been observed in the early and intermediate stages of tumorigenesis in animal models of carcinogen-induced HCC (Yang et al., 2013). Although high FGF expression may drive the oncogenic FGF-FGFR axis and activate signaling that controls tumor cell growth and survival, FGF21 does not appear to stimulate cell proliferation despite the potential activation of mitogen-activated protein kinase (MAPK) signaling. The exact role of FGF21 in hepatocarcinogenesis remains largely unknown.

In this study, we found that gene expression of the E3 ubiquitin-protein ligase TRIM31 is upregulated by HCV infection and that its upregulation is canceled by the knockdown of FGF21 (Table 2, Figure 8A). It has been shown that TRIM31 expression is significantly higher in HCC tissues than in distal non-cancerous liver tissues of HCC patients, and TRIM31 overexpression possibly correlates with HCC progression (Guo et al., 2018; Wang et al., 2023), and that TRIM31 contributes to the activation of the mTORC1 pathway by promoting the ubiquitin-dependent degradation of the TSC1-TSC2 complex, an upstream repressor of the mTORC1 pathway (Guo et al., 2018). Consistent with this finding, we found that the steady-state level of TSC2 is decreased by HCV infection in an infectious titer-dependent manner (Figure 8D) and that the decrease in TSC2 level due to HCV infection is canceled by knockdown of FGF21 (Figure 8C). The mTORC1 signaling pathway regulates cell growth and proliferation by sensing fluctuations in nutrients and growth factors under environmental conditions, mediating the balance between anabolism and catabolism (Szwed et al., 2021). mTORC1 signaling is a major tumorigenic pathway in HCC (Bhat et al., 2013) and is upregulated in 40–50% of HCC and plays an important role in the development and progression of HCC (Lu et al., 2021). The FGF21 expression-dependent upregulation of TRIM31 induced by HCV infection, leading to activation of the mTORC1 signaling pathway, may contribute to the molecular mechanism of hepatocarcinogenesis from HCV infection.

In this study, we also found SOCS2 is one of the genes whose upregulation by HCV infection depends on FGF21 (Table 2, Figure 7A). Members of the SOCS family are cytokine-inducible negative regulators of cytokine receptor signaling via the JAK-STAT pathway and are the main regulators of the innate immune response induced by microbial pathogens. SOCS2 is known as a key negative regulator of the JAK-STAT pathway. Although it has been previously reported that SOCS2 gene expression is upregulated by HCV infection (Blackham et al., 2010), the molecular mechanism was unknown. In this study, we confirmed the upregulation of SOCS2 expression by HCV infection and further demonstrated that FGF21 is involved in its induction mechanism.

The ISRE reporter assay indicated that the activation of IFN signaling is greatly reduced by FGF21 knockdown (Figure 7C). Our microarray analysis revealed that SOCS2 expression was upregulated 2.6-fold by the addition of tunicamycin, suggesting that the ER stress-related signaling pathway is involved in the transcriptional upregulation of SOCS2. In the SOCS family, SOCS1, SOCS2, SOCS3, and CIS are considered to be known to inhibit the type 1 interferon signaling pathway. All of them are expressed to a certain extent in liver tissues, with SOCS2 being most significantly upregulated by HCV infection (Figure 7A).

It may be likely that FGF21 induced via ER stress in HCV infection negatively regulates the innate immune response by upregulating SOCS2 expression, contributing to persistent viral infection. While FGF21 released from hepatocytes potentially acts in an endocrine, autocrine, or paracrine manner to regulate ketogenesis, glycogenesis, and fatty acid oxidation, the molecular mechanisms of FGF21-dependent expression of their regulatory factors are not always clear. Further detailed molecular analyses are needed to clarify how the FGF21 signaling system is linked to the transcriptional regulation of SOCS2 and TRIM31.

It should be noted that according to the gene sequence search, the promoter regions of SOCS2 and TRIM31 do not contain ATF4 recognition sequences. It is also unclear why the induction of SOCS2 mRNA expression by HCV infection is more pronounced than that of SOCS1, SOCS3, and CIS. We plan to investigate the molecular mechanisms of SOCS2 promoter activation by HCV infection, both FGF21-dependent and FGF21-independent. Based on the findings obtained, further comparative analyses of the induction mechanism of SOCS2 expression and the transcriptional regulation of SOCS1, SOCS3, and CIS should be conducted. This series of analyses is expected to provide a complete characterization of the induction of SOCS2 expression by HCV infection.

In addition to TRIM31 and SOCS2, RNA-seq analysis in this study identified several other factors involved in cell proliferation control and immune regulation as genes whose expression is induced by HCV and significantly reduced by FGF21 knockdown (Table 2). Although hepatokine FGF21 possesses multiple physiological functions, its role in regulating cell proliferation and immune mechanisms is relatively unknown compared to its metabolic regulatory actions. Elucidating the effects of increased expression of the factors listed in Table 2 on hepatocyte proliferation and immune response to infection will help us understand the role of FGF21 in the pathogenesis of HCV-related diseases.

## Data availability statement

The datasets presented in this study can be found in online repositories. The RNA-seq data presented in the study are deposited in the NCBI Sequence Read Archive (SRA) repository, accession number SAMN41994644, SAMN41994645, SAMN41994646, and SAMN41994647. The microarray data presented in the study are deposited in the Gene Expression Omnibus (GEO) repository, accession number GSE270921.

## Author contributions

LL: Data curation, Writing – original draft, Formal analysis, Investigation, Methodology, Software, Visualization. MI: Data curation, Formal analysis, Investigation, Methodology, Resources, Software, Supervision, Validation, Writing – review & editing. SSak: Data curation, Formal analysis, Investigation, Methodology, Software, Supervision, Validation, Writing – review & editing. JL: Investigation, Methodology, Resources, Visualization, Writing – review & editing. KO: Investigation, Methodology, Resources, Validation, Writing – review & editing. KS: Resources, Writing – review & editing, Investigation, Methodology. KN: Data curation, Formal analysis, Supervision, Validation, Writing – review & editing, Resources. SSat: Data curation, Formal analysis, Supervision, Validation, Writing – review & editing. AK: Data curation, Formal analysis, Supervision, Validation, Writing – review & editing. TS: Conceptualization, Data curation, Formal analysis, Funding acquisition, Project administration, Supervision, Validation, Writing – original draft.
