# Supplementary material for: FGF21 upregulation by hepatitis C virus via the eIF2α-ATF4 pathway: implications for interferon signaling suppression and TRIM31-mediated TSC degradation
Source: Front Microbiol. 2024 Aug 15;15:1456108. doi: 10.3389/fmicb.2024.1456108 (PMC11357932; doi:10.3389/fmicb.2024.1456108)
Supplement: Supplementary file 1 [file Data_Sheet_1.PDF]

Supplementary Materials

FGF21 Upregulation by Hepatitis C Virus via eIF2α-ATF4 Pathway: Implications for Interferon Signaling Suppression and TRIM31-Mediated TSC Degradation

Liang Liu, Masahiko Ito, Satoshi Sakai, Jie Liu, Kazuyoshi Ohta, Kenji Nakashima, Shinya Satoh, Alu Konno and Tetsuro Suzuki*

* Address correspondence to Tetsuro Suzuki
Department of Microbiology and Immunology, Hamamatsu University School of Medicine, 1-20-1 Handayama, Chuou-ku, Hamamatsu, Shizuoka 431-3192, Japan
E-mail address: tesuzuki@hama-med.ac.jp

Supplementary Figure 1

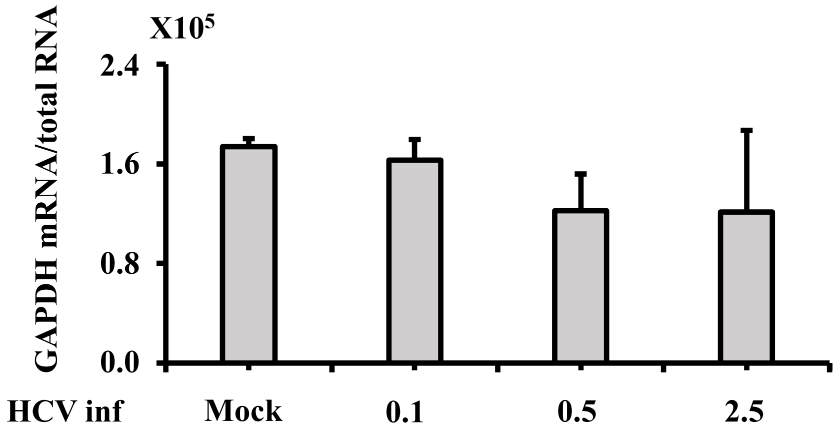

Supplementary Figure 1. In HCV-infected cells (MOI=0, 0.1, 0.5 and 2.5), FGF21 and ATF3 mRNA expression was upregulated as shown in Figure 1A. Using these cellular RNA samples, GAPDH mRNA was determined by RT-qPCR. The final values were normalized to 1ug RNA.

Supplementary Figure 2

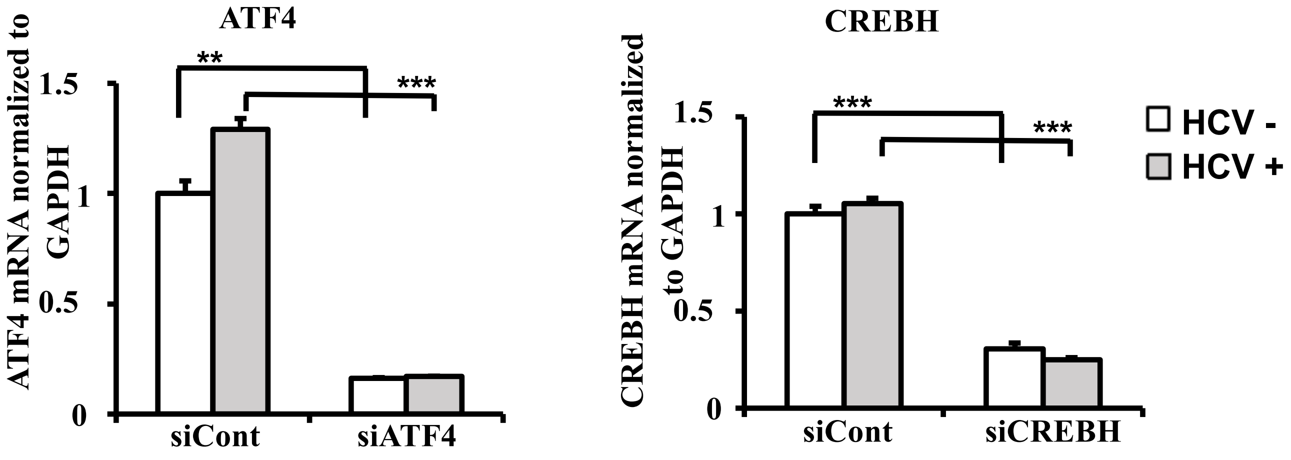

Supplementary Figure 2. Knockdown of CREBH or ATF4 markedly reduced FGF21 mRNA expression as shown in Figure 4A and C. In this experiment, knockdown efficiency of siATF4 and siCREBH was confirmed by RT-qPCR. Results are presented as means ± SD (n=3). Statistical analysis was conducted by ANOVA with post hoc Tukey's test for pairwise group comparisons. **$p < 0.01$; ***$p < 0.001$.

Supplementary Figure 3

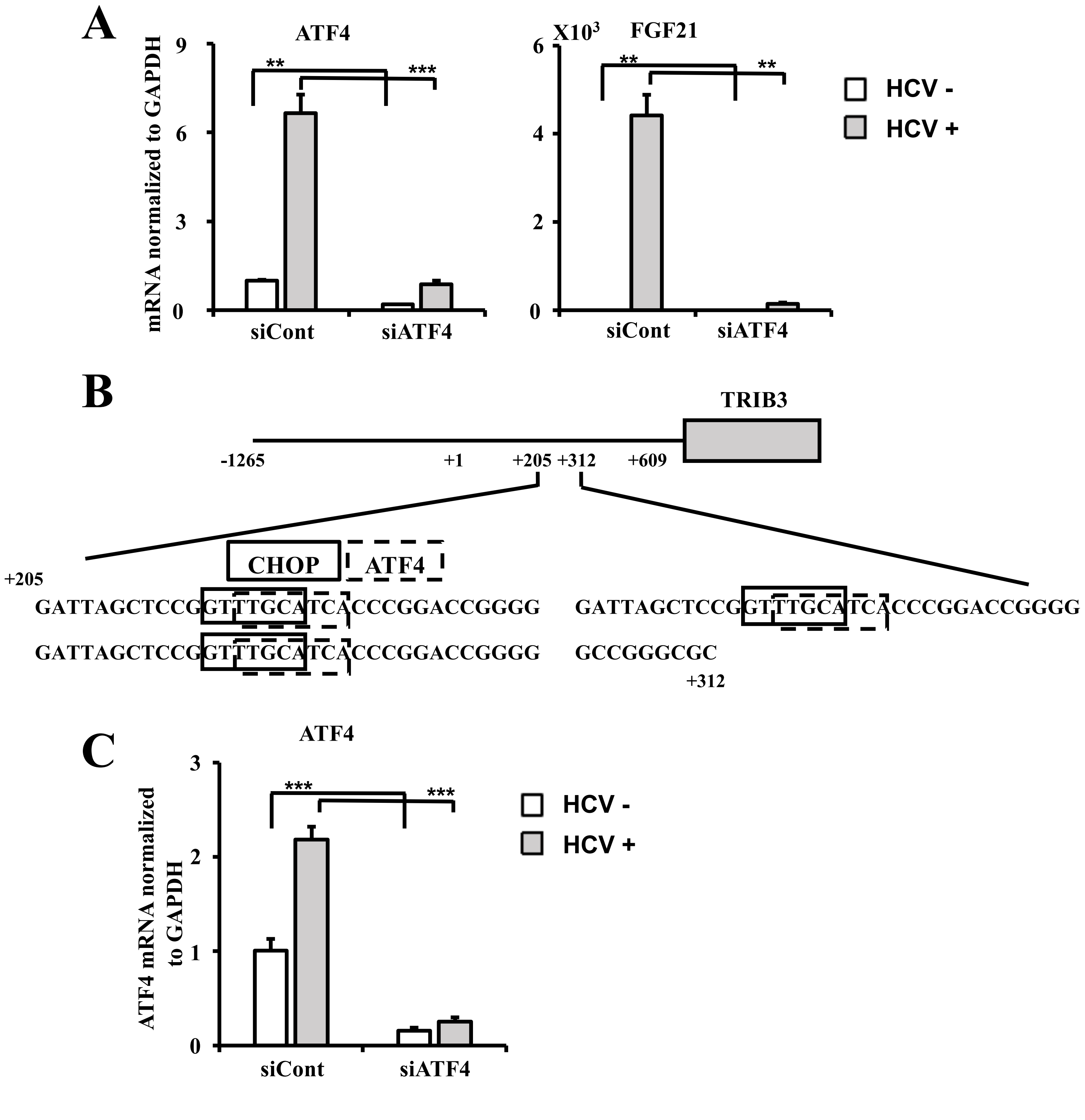

Supplementary Figure 3. (A) Knockdown of ATF4 significantly reduced the expression of TRIB3 mRNA as shown in Figure 5B. In this experiment, the efficiency of siATF4 knockdown and the reduction of FGF21 mRNA expression by ATF4 knockdown were confirmed. mRNA expression levels of ATF4 and FGF21 were determined by RT-qPCR. (B) The schematic representation illustrates the sequence information spanning +205 to +312 in the TRIB3 promoter, highlighting the recognition sites for CHOP and ATF4, respectively. (C) ATF4 knockdown significantly reduced CHOP mRNA expression as shown in Figure 5E. In this experiment, the efficiency of siATF4 knockdown was confirmed. Results are presented as means $\pm$ SD (n=3). Statistical analysis was conducted by ANOVA with post hoc Tukey's test for pairwise group comparisons. **$p < 0.01$; ***$p < 0.001$.

Supplementary Figure 4

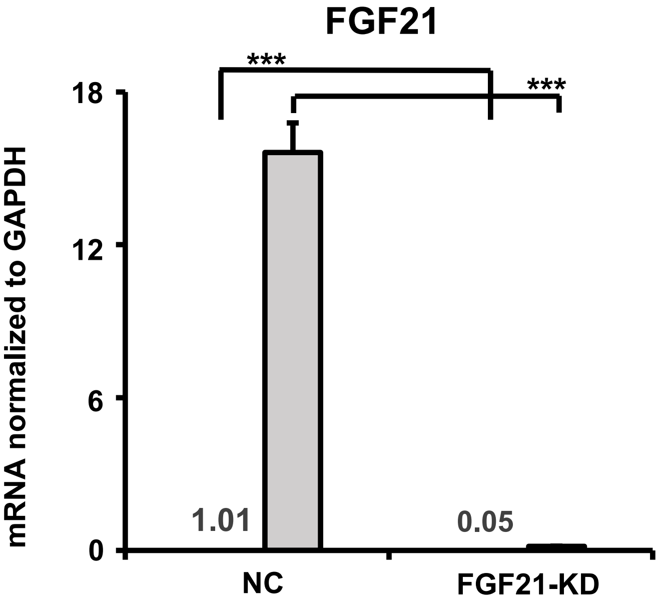

Supplementary Figure 4 FGF21-KD and parental Huh7.5.1 cells for NC were cultured with or without HCV infection for 3 days, followed by determination of mRNA levels of FGF21 were by RT-qPCR. Results are presented as means ± SD (n=3). Statistical analysis was conducted by ANOVA with post hoc Tukey's test for pairwise group comparisons. ***$p < 0.001$.

Supplementary Figure 5

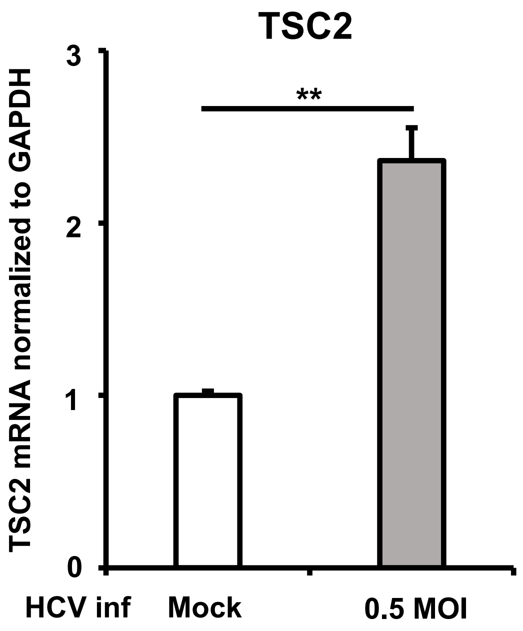

Supplementary Figure 5. Huh7.5.1 cells were cultured with or without HCV infection (MOI=0.5) for 3 days, followed by determination of mRNA levels of TSC2 and GAPDH in cells were by RT-qPCR. Results are presented as means $\pm$ SD (n=3). Statistical analysis was conducted by ANOVA with post hoc Tukey's test for pairwise group comparisons. **p < 0.01.

Supplementary table 1

Primer name	Sequence (5'→3')
pCAGGS core F	GCCTCTGCTAACCATGTTCATGC
pCAGGS core R	GAAGGTGGCGGACATCACAAC
ATF4 F1	CACGGCATTCAGCAGCAGCGTTG
ATF4 R1	TCGCTGCTCAGGAAGCTCATTTCG
pro-M-F1	GATTAAAGTAGGGAGGAGGAGGCTGG
pro-M-R1	CTACTTTAATCCTGGTGTTTCTTTCACCAGAGA
pro-M-F2	TTGGCAAATTGCCAGGTGAGAGG
pro-M-R2	CTGGCAATTTGCCAACCTCAACCTC
GAPDH-F	AACAGCCTCAAGATCATCAGC
GAPDH-R	GGATGATGTTCTGGAGAGCC
FGF21-F	ACCTGGAGATCAGGGAGGAT
FGF21-R	AGTGGAGCGATCCATACAGG
chip-ATF4(-458-369)-F	CCCAGGTTATTTCAGGAGCA
chip-ATF4(-458-369)-R	ATGCTCAGACCCTGGACATC
chip-N(-1918-1832)-F	ACGAACTTCAAAGAGGAACCAGGAG
chip-N(-1918-1832)-R	CATCCTGCTGTGCTGTGCTTCAAT
TRIB3 F2	CCGTGAGAGGAAGAAGCTGGTG
TRIB3 R2	TTGCCGAAGAGCAGGACAGG
CERBH-F	CGGATTTAGCTGCTGGAAAG
CREBH-R	AGCTCCACGTGTCTCAGGAT
TRIM31-F1	AGGAGAAGGAGACAGTACAAGTGAAGG
TRIM31-R1	ATAGCAGGAAATTCTTCTCCTCCTC
SOCS2-F2	CCATTATGTCAAAGGTCCAGGCTCC
SOCS2-R2	GGCCTGTGTCAGCTTGGTTC
SOCS3-F1	AGCTGGTGGTGAACGCAGTG
SOCS3-R1	GAGCTGTCGCGGATCAGAAAGG
SOCS1-F1	CAGAACCTTCCTCCTCTTCCTCCTC
SOCS1-R1	AGTAGAATCCGCAGGCGTCC
CISH-F2	GGGAGGATGACATGCAGAGGAAC
CISH-R2	AGAGAAGCCAGGGCAGAGTTG
HCV-F	GAGTGTCGTGCAGCCTCCA
HCV-R	CACTCGCAAGCACCCTATCA

Supplementary table 1. Primer sequences used in this study.
